# Sodium Alginate/Chitosan/Activated Carbon Composite Hydrogel for Cyanobacterial Inhibition: RSM Optimization and Sustained Release Performance

**DOI:** 10.3390/gels12060496

**Published:** 2026-06-03

**Authors:** Dongmei Jiang, Yingjun Wang

**Affiliations:** College of Environment, Sichuan Agricultural University, Chengdu 611130, China

**Keywords:** *Citrus reticulata* peel, algal inhibition, activated carbon, gel microspheres, response surface methodology

## Abstract

This study presents a sodium alginate/chitosan/activated carbon (SA/CS/AC) gel microspheres loaded with *Citrus reticulata* peel allelochemicals for continuous inhibition of *Microcystis aeruginosa* by controlled release. Preparation parameters were optimized via response surface methodology (RSM) for improved algal inhibition, yielding an optimal formulation: 1.97% SA, 0.76% CS, 0.31% AC. The optimized gel microspheres showed a 7-day inhibition rate of 85.17 ± 2.49%, consistent with the predicted 85.29%. Characterization revealed that AC optimized the gel’s porous structure and surface functionality, providing more adsorption sites for allelochemicals. This helps improve the loading capacity of the gel microspheres and enables stable sustained release, with a cumulative release of 70% over 25 days. Algal inhibition declined slightly from day 7 to 30 due to allelochemical depletion but remained 76.27%, versus 30.58% for the blank SA/CS/AC carrier and 52.81% for the allelochemical-loaded SA/CS gel microspheres. AC thus synergistically strengthens algal inhibition by elevating allelochemical loading and prolonging activity, providing a feasible strategy for sustainable cyanobacterial bloom control.

## 1. Introduction

In recent years, the loads of agricultural non-point source pollution and urban anthropogenic pollution have remained at high levels. Water eutrophication and climate change have led to frequent cyanobacterial blooms. Among them, cyanobacterial blooms dominated by *Microcystis aeruginosa* [1] not only cause water quality deterioration, disruption of aquatic ecosystems, impairment of fisheries, and threats to drinking water safety, but also result in the release of microcystins, which pose a serious threat to human health through the food chain [2,3]. Traditional physical and chemical algal control methods are limited by high cost and the risk of secondary pollution [4], which can hardly meet the requirements of green and long-term remediation. In contrast, plant allelopathy-based algal inhibition technology has gradually emerged as one of the most concerned research directions in green algae control due to its advantages of wide availability, biodegradability, low non-target impact, and low treatment cost [5].

Plant-derived allelochemicals are natural secondary metabolites that can effectively inhibit algal growth after being released into the environment [6]. Among them, phenolic compounds, particularly phenolic acids and flavonoids, are the focus of green algal inhibition research due to their wide distribution and remarkable algicidal activity [7]. The primary allelochemicals in *Citrus reticulata* peel extract (phenolic acids and flavonoids) suppress cyanobacterial growth by triggering ROS-mediated oxidative stress, impairing photosystem II function and chlorophyll synthesis, disrupting membrane integrity, and interfering with core metabolic and gene expression pathways [8,9]. In addition to tissues from terrestrial and aquatic plants, various agricultural by-products such as fruit peels have also been verified to be rich in allelochemicals [10]. Relevant research has shown that the peels of sour orange, grapefruit, and *Citrus reticulata* (including satsuma mandarin) are rich in allelochemicals [11], particularly flavonoids and phenolic acids, with total phenolic contents ranging from 16 to 50 mg GAE/g DW [12], which are substantially higher than those of straw [13], rice husks [14], and forage [15] (1–20 mg GAE/g DW). The aqueous *Citrus reticulata* peel extract exhibits a 7-day inhibition rate of up to 90% against *Microcystis aeruginosa* [16], making it an ideal raw material for the development of natural algicides. Therefore, *Citrus reticulata* peel was selected as the source of allelochemicals in this study. However, natural allelochemicals generally exhibit inherent limitations, including poor stability, easy photodegradation and oxidation, and short efficacy duration [17], which severely limit their practical use. The construction of controlled-release systems using biocompatible hydrogels as carriers can effectively overcome the above bottlenecks, prolong the action period of allelochemicals, and enhance the long-term algal inhibition efficiency [18], representing an important direction for the development of green and long-lasting algicides.

Hydrogels, as polymer carriers with a three-dimensional network structure, exhibit unique advantages for allelochemical loading and sustained release, which can effectively improve the stability of allelochemicals and prolong the algal inhibition [19]. Among various gel carriers, sodium alginate (SA) and chitosan (CS) are two typical natural polymers with outstanding advantages including non-toxicity, good biocompatibility, and biodegradability [20,21]. An interpenetrating network structure can be constructed between them via electrostatic interactions, and SA is capable of ionic crosslinking under the induction of divalent cations such as Ca^2+^, ultimately forming a structurally stable three-dimensional network hydrogel [22,23]. SA/CS hydrogels have emerged as ideal carriers for allelochemical loading owing to their biocompatibility and sustained-release properties. Ni [24] et al. encapsulated artemisinin in SA/CS hydrogels, extending the algae-inhibiting period to over 40 days, which confirms the application potential of such carriers in long-acting algae inhibition.

However, pristine SA/CS hydrogels still exhibit limitations in adsorption performance, mechanical strength, thermal stability, and swelling behavior. Composite fabrication with carbon-based materials is an effective optimization approach [25]. Typical carbon-based materials such as biochar [26], graphene [27] and carbon nanotubes [28] have been widely used in hydrogel modification due to their excellent structure and properties. As reported by He et al. [29], the SA-KBC-Fe/La composite hydrogel possesses a maximum phosphate adsorption capacity that is threefold higher than that of the biochar-free counterpart. Among them, activated carbon (AC) as a typical carbonaceous material, not only possesses a high specific surface area, well-developed hierarchical pores and abundant surface oxygen-containing functional groups [30], but also can directly interfere with the physiological activities of algal cells and exert an algal inhibition effect through its surface chemical properties (e.g., persistent free radicals, C and O content) [31]. These studies indicate that the introduction of AC can effectively improve the material properties and algal inhibition effect of SA/CS gel microspheres, and is expected to enhance their loading capacity for allelochemicals.

Current research on slow-release anti-algal materials mainly focuses on artificially synthesized allelochemicals. Research on composite microspheres based on natural *Citrus reticulata* peel extracts remains limited and lacks systematic investigation. This study innovatively combines natural multi-component allelochemicals with activated carbon gels to construct an integrated slow-release anti-algal system. Compared with conventional SA/CS hydrogels, this system reduces ecological risks via natural allelochemicals, uses activated carbon to optimize microsphere structure and increase contact sites, and enables *Citrus reticulata* peel waste valorization. Response surface methodology was employed to optimize the formulation and systematically characterized the microsphere structure and release behavior. It also compared anti-algal performance with appropriate controls and assessed whether AC improves the carrier system in a meaningful way. This work offers new materials and strategies for the eco-friendly control of cyanobacterial blooms.

## 2. Results and Discussion

### 2.1. LC-MS Identification of Core Allelochemicals in Citrus reticulata Peel Extract

Qualitative analysis was performed using ultra-high-performance liquid chromatography-quadrupole time-of-flight tandem mass spectrometry (UHPLC-QTOF-MS/MS). The system was operated in negative electrospray ionization (ESI^−^) mode. Chemical structures were confirmed by MS/MS fragmentation pattern analysis and database matching (matching score ≥ 80, mass-to-charge ratio deviation < 1 ppm), with detailed chromatographic conditions provided in Section 4.2.2. Detailed LC-MS characterization data for allelochemicals in *Citrus reticulata* peel are provided in the Supplementary Materials (Figures S1–S21).

As shown in Table 1, 20 common allelopathically active compounds were detected, with 19 phenolic substances (dominant components) including phenolic acids (e.g., benzoic acid, cinnamic acid) and flavonoids.

Based on the LC-QTOF-MS/MS analysis, three groups of components were separated according to polarity. The strongly polar components were eluted within 0.5–4 min, mainly including organic acids and small phenolic acids. Medium-polar components were eluted at 4–7 min, which were dominated by flavone glycosides, with hesperetin as the most abundant constituent. Weakly polar components were eluted at 7–16 min, mainly composed of flavone aglycones and fatty acids.

Due to their stronger polarity, phenolic acids generally exhibit shorter retention times than flavonoids on reversed-phase C18 columns. Their tandem mass spectrometry fragmentation is dominated by the loss of neutral small molecules, and the fragmentation patterns are closely related to their skeleton types, as well as the types and numbers of substituents [32]. For phenolic acids devoid of methoxyl substituents (e.g., protocatechuic acid [33], p-coumaric acid, 4-hydroxybenzoic acid [34]), the dominant fragmentation mechanism proceeds through C–O bond scission in the carboxyl moiety, leading to the elimination of CO_2_ (44 Da). Subsequent secondary fragmentations encompass the dehydration of phenolic hydroxyl groups (H_2_O, 18 Da) and cyclization-induced CO loss (28 Da). The fragmentation behavior of methoxyl-substituted phenolic acids varies with substitution patterns: monomethoxylated derivatives (e.g., vanillic acid, ferulic acid) preferentially eliminate a methyl radical (•CH_3_) under ESI^−^ mode, followed by CO_2_ loss; dimethoxylated derivatives (e.g., syringic acid, sinapic acid) follow a similar pathway, with further subsequent loss of methoxy radical (–OCH_3_) or formaldehyde (CH_2_O) [35].

Neoeriocitrin and kaempferol 3-O-sophoroside produced stable deprotonated molecular ions [M–H]^−^ at m/z 595.1677 and 609.1813, respectively, in ESI^−^ mode. Upon collision-induced dissociation, both underwent preferential glycosidic bond cleavage, losing a rhamnosyl-glucosyl moiety (308.1107 Da) and a sophorosyl moiety (308.0743 Da), respectively, yielding distinct aglycone-derived diagnostic fragment ions at m/z 287.0561 and 301.0712. Further secondary fragmentation occurred with the loss of neutral small molecules, including CO (27.9949 Da) for neoeriocitrin and CO (27.9949 Da) plus C_3_O_2_ (68.0002 Da) for kaempferol 3-O-sophoroside, producing fragments such as m/z 567.1709 and m/z 286.0481. These fragmentation behaviors are consistent with the typical pattern of flavonoid glycosides, characterized by preferential glycosidic bond cleavage followed by sequential neutral loss from the aglycone [36]. The other five flavonoid compounds, namely naringin, neohesperidin, quercetin 7-rhamnoside, hesperetin, and isoquercitrin, were tentatively identified based on accurate mass measurements (mass deviation < 5 ppm), molecular formula matching, and database searching. Their elution order followed the structure-retention relationship of flavonoids, with more polar glycosides eluting first and less polar aglycones eluting later [37], supporting the reliability of the tentative identification. Flavonoid glycosides exhibited obvious gradient elution characteristics due to the difference in glycosyl substituents (Table 1). Neoeriocitrin (disaccharide substitution, 4.927 min) and naringin (disaccharide substitution, 5.370 min) were eluted earlier than quercetin 7-rhamnoside (monosaccharide substitution, 5.916 min), indicating that the increase in glycosyl number leads to stronger polarity and shorter retention time on reversed-phase C18 column.

### 2.2. Optimization and Interpretation Based on Response Surface Methodology

#### 2.2.1. Experimental Data Basis for Response Surface Optimization

A three-factor, three-level response surface methodology (Box–Behnken design) was employed with algal inhibition rate as the response value. The independent variables were SA concentration (A), CS concentration (B), and AC concentration (C) (Table 2). The algal inhibition rates of the 17 runs ranged from 66.74% to 86.55%. All repeated central point experiments exhibited inhibition rates above 83%, indicating good experimental stability. A regression model was constructed via Design-Expert to link dosing concentrations to algal inhibition rate (Y).

(1) $$Y=85.21-0.52\times A+0.64\times B+1.61\times C+1.35\times AB+1.77\times AC-3.51\times BC-2.55\times A^{2}-3.48\times B^{2}-9.68\times C^{2}$$

In this equation, Y represents the algal inhibition efficiency of the gel microspheres material. (*w*/*v*, %). A is the sodium alginate concentration (*w*/*v*, %). B is the chitosan concentration (*w*/*v*, %). and C is the activated carbon concentration (*w*/*v*, %).

#### 2.2.2. Regression Model Development and ANOVA Significance Analysis

The Analysis of variance (ANOVA) results (Table 3) confirmed that the regression model exhibited extremely high significance (F = 31.16, *p* < 0.0001), with a non-significant lack-of-fit term (*p* = 0.2650). The determination coefficient R^2^ = 0.9756, adjusted R^2^ = 0.9443, Both values were close to 1, indicating high reliability of the model [38]. coefficient of variation = 1.18% (<10%), and adequate precision = 16.8254 (>4), indicating high goodness of fit and reliability for predicting the relationship between preparation parameters and algal inhibition rate. The significant influencing factors (*p* < 0.05) were C, AC, BC, A^2^, B^2^, and C^2^. The order of influence on algal inhibition rate was: AC content (C) > CS content (B) > SA content (A). Activated carbon, chitosan, and sodium alginate all exerted significant effects on algal inhibition, with strong interactions observed, most notably between chitosan and activated carbon. The quadratic regression model was effective for investigating and identifying the optimum formulation parameters of the SA/CS/AC gel microspheres. All response surface modeling and ANOVA analyses in this section were performed with Design-Expert 13, with further details provided in Section 4.6.

#### 2.2.3. Analysis of Response Surface Interactions

2D contour and 3D response surface plots were generated using Design-Expert to evaluate the effects of sodium alginate (A), chitosan (B), and activated carbon (C) concentrations on the algal inhibition rate of SA/CS/AC gel microspheres. Based on the quadratic regression model, interaction effects were analyzed. The slope and curvature of the response surfaces reflect factor effects and interactions, while contour density indicates their relative significance [39].

**Figure 1:**
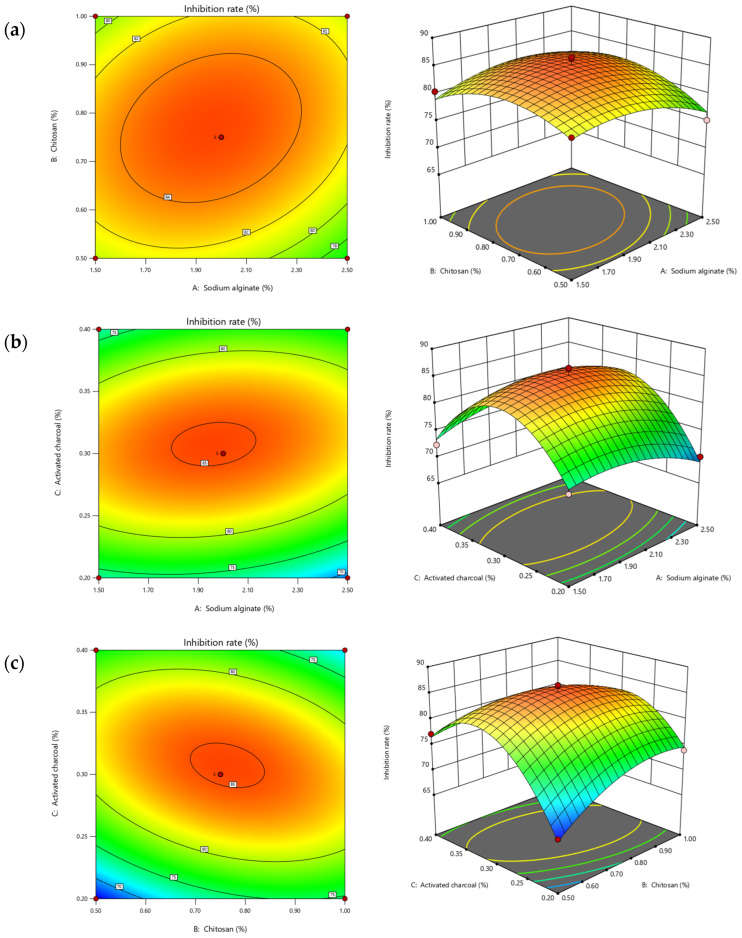
Response surface and contour plots showing the interactive effects of sodium alginate (A), chitosan (B), and activated carbon (C) concentrations on the algal inhibition rate of SA/CS/AC gel microspheres. (**a**) Interaction between A and B; (**b**) Interaction between A and C; (**c**) Interaction between B and C.

As shown in Figure 1, the effects and interactions of sodium alginate (A), chitosan (B), and activated carbon (C) on the algal inhibition rate of the gel microspheres were systematically analyzed by fixing each single factor at the central point level.

When C was fixed at 0.3% (Figure 1a), the response surface showed a gentle slope, and the contour plot was approximately circular with sparse distribution, which was consistent with ANOVA. These results indicated that both the main effects of A and B and their interaction were weak. Nevertheless, the algal inhibition rate first increased and then decreased with increasing A and B concentrations, showing a quadratic response dominated by their significant quadratic terms. Insufficient concentrations led to poor microsphere formation and insufficient active component loading, while excessive concentrations resulted in a dense gel network that hindered the release of active substances, both of which reduced algal inhibition efficiency.

When B was fixed at 0.75% (Figure 1b), the response surface was steeper along the C axis, and the contour lines were typically elliptical and densely distributed along C, visually indicating that the main effect of C was significantly stronger than that of A, with a significant interaction between them. The influence order was C > A. The algal inhibition rate exhibited a quadratic change with increasing A and C concentrations. This nonlinear response was jointly dominated by their significant quadratic and interactive terms. Deviation from the optimal concentration range caused insufficient active component loading, activated carbon aggregation, or structural damage to the gel matrix, thereby significantly reducing the algal inhibition effect. The algal inhibition rate showed a quadratic variation with increasing A and C concentrations. This nonlinear response was governed by their significant quadratic and interactive terms. Deviation from the appropriate concentration range resulted in insufficient loading of active components, activated carbon aggregation, or structural destruction of the gel matrix, thus markedly weakening the algal inhibition performance.

When A was fixed at 2.5% (Figure 1c), the response surface was steep along B and C axes with a distinct peak, and the contour lines exhibited an elliptical shape with an inclined major axis, confirming a strong synergistic interaction between B and C. The contour lines were more densely distributed along the C axis, indicating that the effect of C was significantly stronger than that of B. The extreme concentration regions corresponded to the optimal balance between adsorption capacity and sustained-release performance.

Overall, the order of influencing factors on algal inhibition rate was: activated carbon content > chitosan content > sodium alginate content.

#### 2.2.4. Optimization of Preparation Parameters and Model Validation

**Figure 2:**
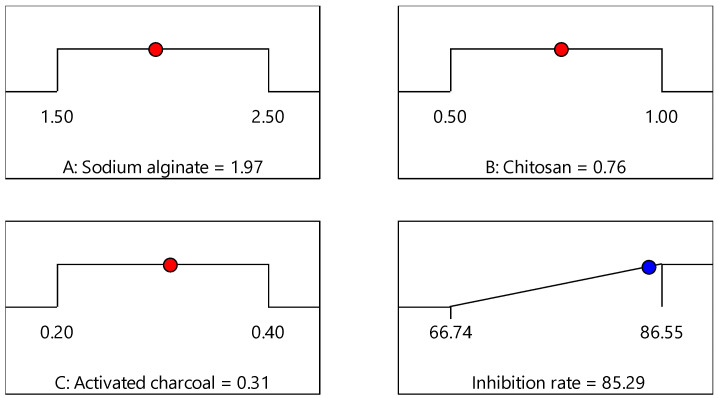
Optimal formulation concentrations and responses via response surface optimization.

The optimal preparation parameters of SA/CS/AC gel microspheres were obtained by response surface methodology using Design-Expert software, and the results are shown in Figure 2. Figure 2 displays the desirability ramp plots of three independent variables (A: sodium alginate, B: chitosan, C: activated carbon) and the corresponding response (algal inhibition rate). The red dot on each ramp represents the optimal level of each factor for maximizing the inhibition rate. The optimal conditions were 1.97% SA, 0.76% CS and 0.31% AC, all within the designed factor ranges, with a predicted inhibition rate of 85.29%. The actual inhibition rate measured from three validation experiments was 85.17 ± 2.49%, which agreed well with the predicted value and confirmed the reliability of the regression model.

**Figure 3:**
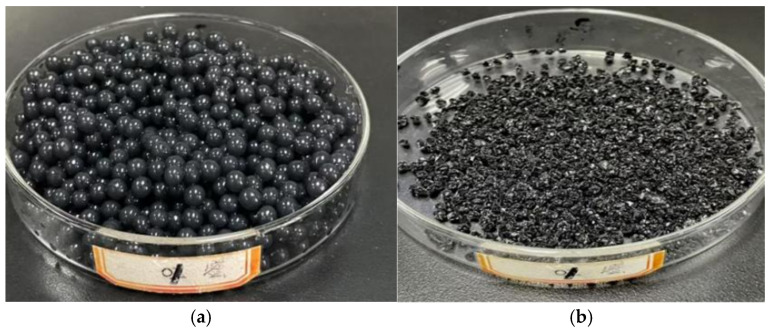
Morphology of SA/CS/AC composite gel microspheres before (**a**) and after (**b**) drying under optimal preparation conditions.

Figure 3 shows the macroscopic morphology of the SA/CS/AC composite gel microspheres before (a) and after (b) freeze-drying, respectively. The final product is grey-black, loosely porous microspheres with uniform particle sizes ranging from 1 to 2 mm. In the algal inhibition assay, the composite gel microspheres remained as solid particles. After absorbing water, most microspheres gradually settled to the bottom, while a small fraction stayed suspended in the algal medium.

### 2.3. Release Curve of the SA/CS/AC Gel Microspheres Material

**Figure 4:**
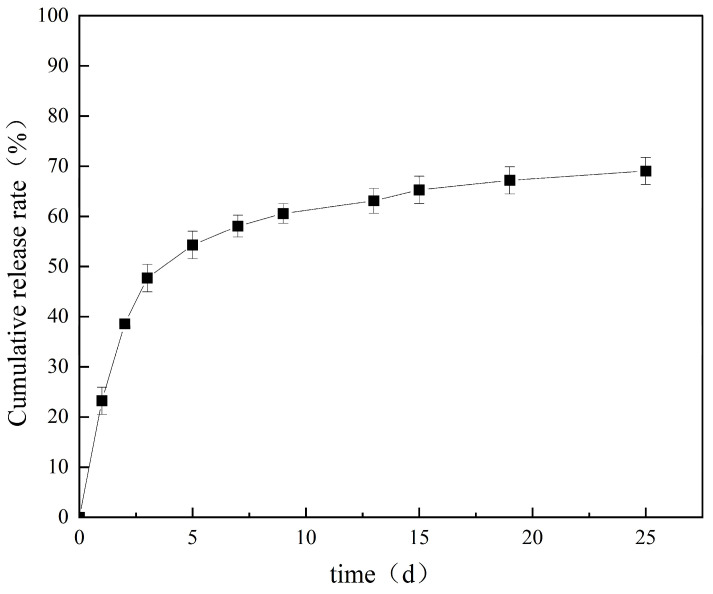
Cumulative release curve of the SA/CS/AC composite gel material.

The release performance of the composite gel material is a core indicator for evaluating its environmental application potential, which directly determines the utilization efficiency and ecological safety of allelochemicals. The release profile presented in this section was obtained using the optimized SA/CS/AC gel microspheres, with the optimal formulation of 1.97% sodium alginate, 0.76% chitosan, and 0.31% activated carbon. The standard curve for total phenolics in *Citrus reticulata* peel was y = 0.0514x + 0.0165 (R^2^ = 0.9723), which exhibited a good linear relationship within the experimental concentration range, indicating that ultraviolet spectrophotometry is effective for determining allelochemical content in *Citrus reticulata* peel. Cumulative release of *Citrus reticulata* peel allelochemicals from the SA/CS/AC microspheres was measured over 25 days (Figure 4), and the release curve is shown below.

The allelochemical release from the gel microspheres exhibited a biphasic characteristic of “rapid release–sustained release”: the first 0–5 days corresponded to the rapid release phase, where the surface-adsorbed allelochemicals were rapidly desorbed, achieving a cumulative release rate of 55% to quickly inhibit algal proliferation; the period from 5 to 25 days was the sustained release phase, during which the allelochemicals slowly diffused through the porous structure of the gel microspheres, with the cumulative release rate reaching 70%, realizing long-term algal inhibition. As the diffusion path of allelochemicals within the SA/CS/AC gel microspheres lengthened and the mass transfer resistance of the polymer matrix increased over time, the release rate gradually decelerated and eventually plateaued, reaching a cumulative release of approximately 70% by day 25.

This release profile suggests the favorable sustained-release characteristics of the gel microspheres. It not only achieves immediate algal inhibition via the initial rapid release but also maintains an effective concentration of allelochemicals in the water through the subsequent stable release. This prolongs the duration of efficacy and avoids the drastic concentration fluctuations typically associated with direct application.

### 2.4. Materials Characterization

#### 2.4.1. SEM Characterization of the SA/CS/AC Gel Microspheres

**Figure 5:**
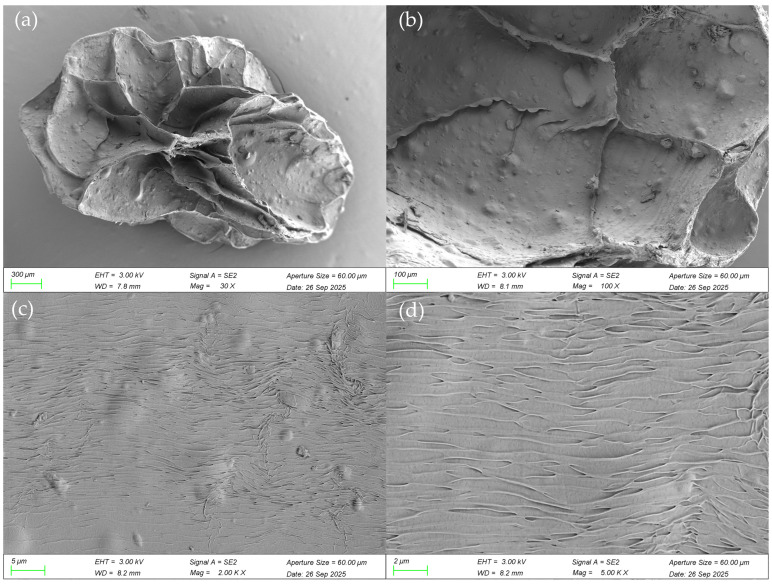
SEM micrographs of the optimized SA/CS/AC gel microspheres at different magnifications. (**a**) 30× magnification; (**b**) 100× magnification; (**c**) 2000× magnification; (**d**) 5000× magnification.

Scanning electron microscopy was used to characterize the micromorphology of the optimized SA/CS/AC gel microspheres (1.97% sodium alginate, 0.76% chitosan, 0.31% activated carbon), with the results shown in Figure 5. As observed in Figure 5a,b (magnification: 30× and 100×, respectively), the SA/CS/AC composite exhibits an irregular ellipsoid-like morphology with evident surface collapse, wrinkling, and local lamellar delamination. In the hydrated state, the hydrogel forms a three-dimensional network through Ca^2+^-mediated ionic crosslinking of sodium alginate (“egg-box” structure), electrostatic interactions between SA and CS, and hydrogen bonding among polymer chains, while activated carbon particles are dispersed throughout the polymer matrix [22,23]. Water removal during freeze-drying induces contraction of the polymer chains and partial collapse of the network structure, resulting in a relatively compact or partially non-porous morphology [40]. At higher magnifications (Figure 5c,d, 2000× and 5000×, respectively), distinct interlayer gaps and porous channels are observed. The combined structure of surface wrinkles, stacked lamellae, and interlayer gaps is conducive to increasing the interfacial contact area of the microspheres, and may facilitate the diffusion and mass transfer of allelochemicals [41]. The current observations corroborate the conclusions reported in the study by Huang and colleagues [42].

#### 2.4.2. Functional Groups of SA/CS/AC Gel Microspheres

**Figure 6:**
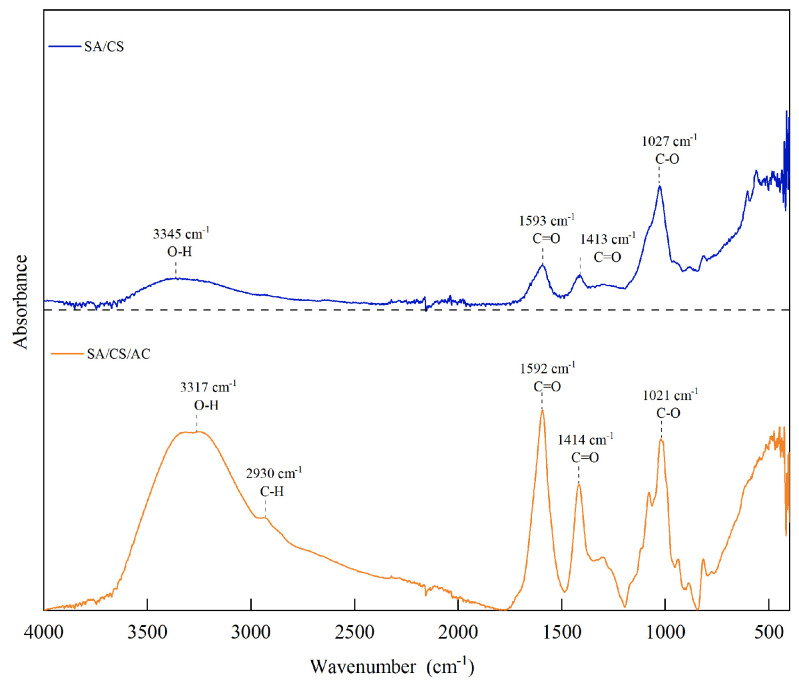
Infrared spectra of SA/CS gel microspheres and SA/CS/AC gel microspheres.

As illustrated in Figure 6, broad absorption bands are observed at 3345 cm^−1^ and 3317 cm^−1^ in the spectra of both SA/CS gel microspheres and SA/CS/AC gel microspheres. These peaks come from –OH and –NH stretching vibrations, indicating hydrogen-bonding networks in the gel microspheres matrix [43]. Compared to SA/CS, this band shifts slightly to a lower wavenumber (3317 cm^−1^) in SA/CS/AC, which points to stronger hydrogen bonding after adding activated carbon. Distinct absorption features at ~1593 cm^−1^ and 1413 cm^−1^ arise from the asymmetric and symmetric stretching vibrations of carboxyl groups (COO^−^), respectively. The 1027 cm^−1^ and 1021 cm^−1^ bands are attributed to C–O stretching and C–O–C linkages in the polysaccharide backbone [44]. These characteristic peaks are retained in the composite, indicating that the fundamental structure of SA/CS remains intact. A new peak at 2930 cm^−1^, assigned to C–H stretching, appears in SA/CS/AC. The intensities of the 1592 cm^−1^, 1414 cm^−1^, and 1021 cm^−1^ bands also increase, likely due to activated carbon and its surface oxygen-containing groups [45]. These results indicate that the addition of activated carbon alters the surface chemical environment of the gel microspheres, which is beneficial for the adsorption and slow release of *Citrus reticulata* peel allelochemicals.

### 2.5. Algae Inhibition Experiments of SA/CS/AC Gel Microspheres

**Figure 7:**
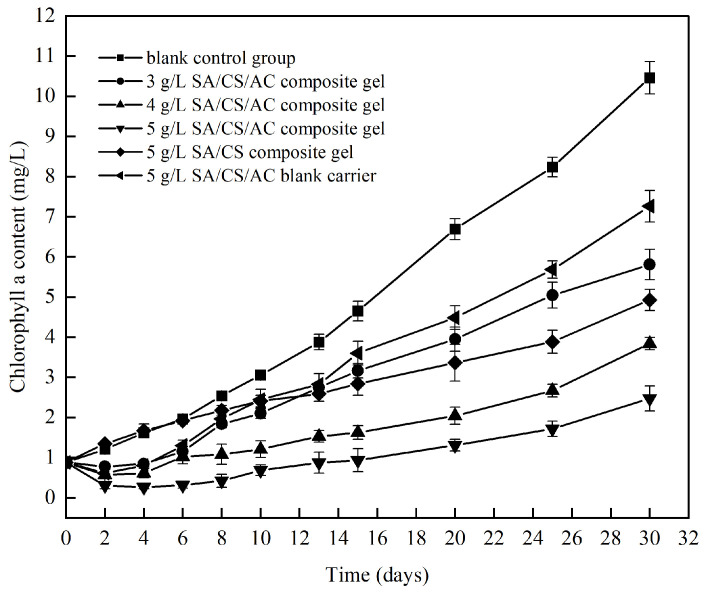
Effects of blank SA/CS/AC carrier, SA/CS gel microspheres, and SA/CS/AC gel microspheres on the chlorophyll-a content in *Microcystis aeruginosa* cells. Note: Blank SA/CS/AC carrier refers to the gel microspheres without loading *Citrus reticulata* peel allelochemicals. SA/CS and SA/CS/AC gels are loaded with allelochemicals from *Citrus reticulata* peel.

Chlorophyll-a (Chl-a) concentration acts as a pivotal metric for algal photosynthetic activity, enabling indirect assessment of algal physiological state and viability [46]. Changes in Chl-a content of *Microcystis aeruginosa* under different treatments are shown in Figure 7. The results indicate that, compared with the blank control, SA/CS/AC gel microspheres at all tested concentrations (3–5 g/L) significantly inhibited Chl-a accumulation (*p* < 0.05), and the inhibitory effect increased with increasing material concentration. Algal inhibition decreased slightly from day 7 to 30, owing to sustained allelochemical consumption and limited loading, which lowered the effective concentration. Nevertheless, the 5 g/L group still maintained a 76.27% inhibition rate on day 30, with chlorophyll-a content of only 2.48 mg/L (23.67% of the control). However, the maximum inhibition rate of the carbon-free control group (SA/CS gel microspheres) was only about 52.81%. The blank SA/CS/AC carrier (without loading *Citrus reticulata* peel allelochemicals) reduced the chlorophyll concentration of *Microcystis aeruginosa* by 30.58%, confirming the intrinsic algal inhibition activity of AC. Abundant persistent free radicals and oxygen-containing surface functionalities (e.g., −OH and C=O) on the AC surface may disrupt the photosynthetic system of *Microcystis aeruginosa*, resulting in reduced chlorophyll a synthesis efficiency, in agreement with previous studies [47,48]. After loading allelochemicals, the algal inhibition rate of the SA/CS/AC gel microspheres further increased to 76.27%, indicating that the intrinsic inhibition of AC and the chemical inhibition of *Citrus reticulata* peel allelochemicals generated a physico-chemical synergistic effect that significantly enhanced the algal inhibition performance.

In practical terms, the SA/CS/AC microspheres made in this study have some potential for real eutrophic water. Their slow release extends algal inhibition. Made from natural materials, they pose little risk of secondary pollution. They offer a practical option for controlling cyanobacterial blooms.

## 3. Conclusions

In this study, we successfully prepared a SA/CS/AC gel microspheres loaded with allelochemicals from *Citrus reticulata* peel, and applied it to inhibit *Microcystis aeruginosa*. Preparation parameters were optimized using Box–Behnken design and response surface methodology. The optimal mass fractions of SA, CS, and AC were determined to be 1.97%, 0.76%, and 0.31%, respectively. Experimental validation confirmed that the regression model was reliable and exhibited satisfactory predictive power. The gel microspheres exhibited a typical rapid initial release followed by slow release. The gel microspheres released allelochemicals quickly in 0–5 days and steadily up to 25 days. Around 70% was released over 25 days. SEM and FT-IR analyses showed that the SA/CS/AC gel microspheres had a well-developed porous structure and abundant surface functional groups, providing a favorable structural basis for loading *Citrus reticulata* peel allelochemicals. Due to the continuous consumption of allelochemicals and limited loading capacity, the algal inhibition rate decreased slightly from day 7 to day 30. However, at a dosage of 5 g/L, the inhibition rate still remained above 76% after 30 days. The algal inhibition rates of blank SA/CS/AC carrier (without allelochemicals) and the AC-free SA/CS gel microspheres were only 30.58% and 52.81%, respectively. This shows that incorporating activated carbon into the carrier together with *Citrus reticulata* peel allelochemicals can significantly improve algal inhibition efficiency. This study provides a feasible and eco-friendly technical approach for the sustainable control of cyanobacterial blooms. However, several limitations remain in the present study. Comparative release experiments using allelochemical extracts were not included, and the swelling behavior and long-term physical and chemical stability of the hydrogel microspheres were not systematically evaluated. Therefore, further studies are needed to better elucidate the release mechanism of the SA/CS/AC hydrogel system and evaluate its stability under practical environmental conditions. Future work will focus on quantitative swelling analysis, comparative release kinetics, and long-term stability assessment to gain deeper insight into the sustained-release behavior of the hydrogel system and support its potential practical environmental applications.

## 4. Materials and Methods

### 4.1. Materials

Fresh *Citrus reticulata* peels were obtained from a local market located near the university campus. The *Microcystis aeruginosa* FACHB-912 isolate employed in the present study was sourced from the Freshwater Algae Culture Collection, Institute of Hydrobiology, Chinese Academy of Sciences. BG11 medium for algal cultivation was purchased from Qingdao Haibo Biotechnology Co., Ltd. (Qingdao, China). Sodium alginate (analytical grade) was provided by Sinopharm Chemical Reagent Co., Ltd. (Shanghai, China). Supplementary reagents, including anhydrous calcium chloride, chitosan, activated carbon, acetone, hydrochloric acid, methanol, and glacial acetic acid, were all of analytical quality and procured from Chengdu Kelong Chemical Co., Ltd. (Chengdu, China).

### 4.2. Material Synthesis and Optimization

#### 4.2.1. Preparation of *Citrus reticulata* Peel Extract

*Citrus reticulata* peel was freeze-dried for 24 h, ground, and sieved (100 mesh). Powder extraction was performed with 80% methanol (1:20, *w*/*v*) at 70 °C over a 2 h period. Post-extraction, the suspension was left to cool naturally and then clarified through filtration. The filtrate was stored at 4 °C prior to further analysis.

#### 4.2.2. LC–MS Characterization of *Citrus reticulata* Peel Extracts

Prior to analysis, all samples were filtered through a 0.22 μm membrane to remove particulate impurities. Chromatographic separation was carried out on a UHPLC-Q-TOF system (1290 Infinity II-6546, Agilent Technologies, Santa Clara, CA, USA) with an Eclipse Plus C18 column. The mobile phase consisted of 0.1% formic acid in water (A) and methanol (B) at a flow rate of 0.3 mL/min. The gradient elution program was as follows: 95% A (0–1.5 min), 60% A (1.5–5 min), 5% A (5–17 min), followed by re-equilibration to the initial conditions. The chromatographic separation method in this study was referenced from the previously reported protocol for the analysis of phenolic compounds in *Citrus reticulata* peel extracts [49].

#### 4.2.3. Synthesis of SA/CS/AC Gel Microspheres

The preparation strategy was adapted from a previously reported method by Bustos et al. [50], with modifications to suit the current experimental design.

(1) An appropriate amount of sodium alginate was dissolved under continuous stirring in a 90 °C water bath for 4 h. After cooling, the pH was adjusted to 4 using 1% HCl. The prepared *Citrus reticulata* peel extract and activated carbon were then added sequentially, mixed at a specified volume ratio, and stirred thoroughly to obtain solution A.

(2) Appropriate amounts of chitosan and anhydrous calcium chloride were dissolved in 1% (*w*/*v*) glacial acetic acid and stirred continuously for 4 h to obtain a homogeneous external crosslinking solution (solution B).

(3) Solution A was added dropwise into solution B to form the gel beads. Magnetic stirring was continued for a certain period to allow sufficient interaction between Ca^2+^ and alginate, thereby strengthening the gel network structure.

(4) After dropping, the formed gel beads were quickly rinsed with distilled water, filtered, and freeze-dried using a vacuum freeze dryer (SCIENTZ-10N/A, Ningbo Scientz Biotechnology Co., Ltd., Ningbo, China). Once the cold-trap temperature of the vacuum freeze dryer reached ≤−56 °C, the sample trays were placed into the chamber and pre-frozen for 6–8 h until the samples were completely solidified. The sample trays were then transferred to the upper drying shelf, the bell jar sealed, the system evacuated, and vacuum freeze-drying performed for 48 h. The dried beads were collected for further use. The final product is grey-black, loosely porous microspheres with uniform particle sizes ranging from 1 to 2 mm.

#### 4.2.4. RSM-Based Experimental Optimization via Response Surface Methodology

Optimization of gel microspheres formulation was conducted using a three-factor, three-level Box–Behnken experimental design. The concentrations of sodium alginate (A), chitosan (B), and activated carbon (C) were chosen as the independent experimental factors, with the algal inhibition rate (Y) set as the response variable (Table 4).

### 4.3. Assessment of Total Phenolics in Citrus reticulata Peel Extract

The method reported by Koolaji [51] et al. was adopted with slight modifications. The total phenolic content in *Citrus reticulata* peel extracts and composite gel microspheres was determined via the Folin–Ciocalteu method. Absorbance was measured at 765 nm using a UV–Vis spectrophotometer (UV-3100, MAPADA, Shanghai, China).

(1) Standard curve establishment: A series of gallic acid standard solutions (1, 2, 3, 5, 10 mg/L) were prepared. Each 1 mL aliquot was mixed with 5 mL of 10% Folin–Ciocalteu reagent. After 5 min standing, 4 mL of 7.5% Na_2_CO_3_ solution was added, and the mixture was incubated in darkness for 60 min. Absorbance was recorded at 765 nm.

(2) Linear relationship between extract concentration and total phenolic content: A series of *Citrus reticulata* peel extract solutions at different concentrations were determined following the above procedure.

### 4.4. Material Characterization

SA/CS/AC gel microspheres were characterized using a scanning electron microscope (ZEISS Sigma 360, Carl Zeiss AG, Oberkochen, Germany) to examine their surface morphology and microstructural features. Following complete dehydration by vacuum freeze-drying (cold-trap temperature ≤ −56 °C, pre-freezing for 6–8 h, and drying for 48 h), the samples were mounted on conductive adhesive tape, and sputter-coated with gold under vacuum. SEM imaging was conducted in secondary electron mode at an accelerating voltage of 5 kV. Images were acquired at magnifications ranging from 30× to 50,000×.

SA/CS/AC gel microsphere chemical structure was analyzed by Fourier transform infrared spectroscopy (FTIR, Nicolet iS50, Thermo Fisher Scientific, Waltham, MA, USA). Dried samples were ground with spectroscopic-grade KBr, pressed into pellets, and analyzed over the range of 4000–500 cm^−1^.

### 4.5. Algal Growth Inhibition Experiment

#### 4.5.1. Cultivation of *Microcystis aeruginosa*

Algal cultures were maintained in BG11 medium under controlled laboratory conditions. Cells were grown to the exponential phase at 25 ± 0.5 °C, with a light intensity of 1500–2200 lx and a 12 h light/dark photoperiod to ensure stable growth dynamics.

#### 4.5.2. Algal Growth Inhibition Experiment of SA/CS/AC Gel Microspheres

The prepared SA/CS/AC microspheres were directly added into 250 mL conical flasks containing 150 mL of *Microcystis aeruginosa* culture in the logarithmic growth phase, at final concentrations of 3.0, 4.0 and 5.0 g/L, respectively. To investigate whether the introduction of activated carbon improves the algal inhibition performance of gel microspheres, three control groups were included simultaneously: a 5.0 g/L SA/CS microsphere group, a 5.0 g/L blank carrier group (without active algal-inhibiting components), and a blank control with only algal culture. Each group was set up in triplicate with a 30-day experimental period. The conical flasks were gently shaken manually twice per day to ensure full contact between microspheres and algal cells. Chlorophyll-a content was measured on days 2, 4, 6, 8, 10, 13, 15, 20, 25 and 30 throughout the experiment.

#### 4.5.3. Algal Growth Inhibition Rate

The inhibition rate of *Microcystis aeruginosa* growth by the SA/CS/AC gel microspheres was evaluated based on Chl-a content [52], calculated as:

(2) $$\mathrm{IR}\;=\;\left(1-N_{t}/N_{0}\right)\;\times \;100\%$$

where IR represents the inhibition rate, and Nt and N_0_ represent the chl-a contents (mg/L) in the treatment and blank control groups, respectively.

Chlorophyll-a content was quantified using an acetone extraction procedure combined with spectrophotometric analysis [53]. Absorbance values were recorded at 630, 645, 663, and 750 nm, and the concentration was calculated according to the following equations.

(3) $$C=11.64\left(A_{663}-A_{750}\right)-2.16\left(A_{645}-A_{750}\right)+0.1(A_{630}-A_{750})$$

(4) $$\mathrm{Chl}\text{-}a=\frac{{\mathrm{CV}}_{1}}{V_{2}}$$

A_630_, A_645_, A_663_, and A_750_ are the absorbance values at 630, 645, 663, and 750 nm, respectively. V_1_ and V_2_ denote the volumes (mL) of algal suspension and 90% acetone, respectively. The calculated Chl-a parameter corresponds to the chl-a content, reported in mg/L.

### 4.6. Data Analysis

All analytical measurements were repeated in three independent replicates, and the resultant data are expressed as mean values. Data processing was carried out using Microsoft Excel 2021, while graphical visualization was completed with Origin 2021. Response surface modeling and statistical analysis were conducted using Design-Expert 13.

## Figures and Tables

**Table 1:** LC-MS Qualitative Identification Results of Common Allelochemicals.

Serial Number	Retention Time	Molecular Formula	M/Z	Mass Deviation /ppm	Compound Name	Category	Matching Score
1	0.645	C_7_H_12_O_6_	191.0560	−0.33	D-(-)-Quinic acid	Organic acid	99.86
2	0.732	C_4_H_6_O_5_	133.0142	−0.56	Malic acid	Organic acid	99.9
3	0.849	C_9_H_13_NO_2_	166.0875	0.72	Synephrine	Alkaloid	99.35
4	1.584	C_7_H_6_O_4_	153.0194	0.47	Protocatechuic acid	Phenolic acid	99.24
5	1.994	C_7_H_6_O_3_	137.0243	−0.59	p-hydroxybenzoic acid	Phenolic acid	99.76
6	2.244	C_7_H_6_O_2_	121.0295	−0.17	Benzoic acid	Phenolic acid	99.98
7	2.288	C_8_H_8_O_4_	167.0350	0.14	Vanillic acid	Phenolic acid	98.87
8	2.731	C_8_H_8_O_5_	183.0298	−0.30	Methyl gallate	Phenolic acid	97.08
9	3.823	C_9_H_8_O_3_	163.0400	−0.26	p-Coumaric acid	Phenolic acid	98.01
10	1.332	C_7_H_10_O_5_	173.0455	−0.22	Shikimic acid	Organic acid	98.22
11	4.927	C_27_H_32_O_15_	595.1666	−0.48	Neoeriocitrin	Flavonoid	98.74
12	5.370	C_27_H_32_O_14_	579.1717	−0.45	Naringin	Flavonoid	99.41
13	5.813	C_28_H_34_O_15_	609.1821	−0.68	Neohesperidin	Flavonoid	99.16
14	5.916	C_21_H_20_O_11_	447.0929	−0.89	Quercetin 7-rhamnoside	Flavonoid	97.69
15	6.494	C_16_H_14_O_6_	301.0716	−0.47	Hesperetin	Flavonoid	83.96
16	6.539	C_21_H_20_O_12_	463.0879	−0.68	Isoquercitrin	Flavonoid	95.64
17	6.509	C_27_H_30_O_16_	609.1461	−0.02	Kaempferol 3-O-sophoroside	Flavonoid	99.73
18	2.746	C_10_H_8_O_3_	175.0401	0.07	7-Methoxycoumarin	Coumarins	98.18
19	10.067	C_14_H_28_O_2_	227.2018	0.43	Myristic acid	Fatty acid	99.77
20	10.471	C_18_H_32_O_2_	279.2328	−0.63	Linoleic acid	Fatty acid	97.83

**Table 2:** Design matrix and measured responses for the response surface optimization.

Serial Number	A (%)	B (%)	C (%)	Inhibition Rate (%)
1	2	0.4	0.2	66.74
2	2	1.3	0.2	73.95
3	2.5	0.4	0.3	75.21
4	2	0.7	0.3	84.10
5	2.5	0.7	0.4	76.62
6	2.5	0.7	0.2	70.08
7	2	0.7	0.3	85.12
8	2.5	1.3	0.3	80.29
9	2	1.3	0.4	70.35
10	1.5	0.7	0.2	72.86
11	1.5	1.3	0.3	80.43
12	2	0.7	0.3	83.89
13	2	0.7	0.3	86.37
14	1.5	0.7	0.4	72.34
15	2	0.7	0.3	86.55
16	1.5	0.4	0.3	80.76
17	2	0.4	0.4	77.16

Note: A, B, and C correspond to the concentrations of SA, CS, and AC (%), in that order.

**Table 3:** ANOVA for the quadratic regression model.

Source	Sum of Squares	d/f	Mean Square	F-Value	*p*-Value	
Model	602.18	9	66.91	31.16	<0.0001	Significant
A	2.19	1	2.19	1.02	0.3457	
B	3.32	1	3.32	1.54	0.2540	
C	20.61	1	20.61	9.60	0.0174	
AB	7.32	1	7.32	3.41	0.1074	
AC	12.46	1	12.46	5.80	0.0468	
BC	49.14	1	49.14	22.88	0.0020	
A^2^	27.47	1	27.47	12.79	0.0090	
B^2^	50.97	1	50.97	23.74	0.0018	
C^2^	394.27	1	394.27	183.61	<0.0001	
Residual	15.03	7	2.15			
Lack of Fit	8.91	3	2.97	1.94	0.2650	Not significant
Pure Error	6.12	4	1.53			
Cor Total	617.22	16				
R^2^ = 0.9756; Adjusted R^2^ = 0.9443						
CV = 1.88%; Adeq Precision = 16.8254						

Note: A, B, and C correspond to the concentrations of SA, CS, and AC (%), in that order.

**Table 4:** Experimental variables and coded levels for the Box–Behnken response surface design.

Factor	Code	The Level of Code		
		−1	0	1
Sodium alginate concentration (*w*/*v*, %)	A	1.5	2	2.5
Chitosan concentration (*w*/*v*, %)	B	0.4	0.7	1
activated carbon concentration (*w*/*v*, %)	C	0.2	0.3	0.4

Note: The optimal concentrations of SA, CS and AC for microsphere preparation were determined from response surface optimization results.

## Data Availability

All raw data supporting the findings of this work are accessible from the lead contact author upon request.

## Supplementary Materials

The following supporting information can be downloaded at: https://www.mdpi.com/article/10.3390/gels12060496/s1.
