# Supplementary material for: Sodium Alginate/Chitosan/Activated Carbon Composite Hydrogel for Cyanobacterial Inhibition: RSM Optimization and Sustained Release Performance
Source: Gels. 2026 Jun 3;12(6):496. doi: 10.3390/gels12060496 (PMC13297986; doi:10.3390/gels12060496)
Supplement: Supplementary file 1 [file gels-12-00496-s001.zip › gels-4307686-supplementary.pdf]

Supplementary Information

Section S1. Annotated Total Ion Chromatogram (TIC)

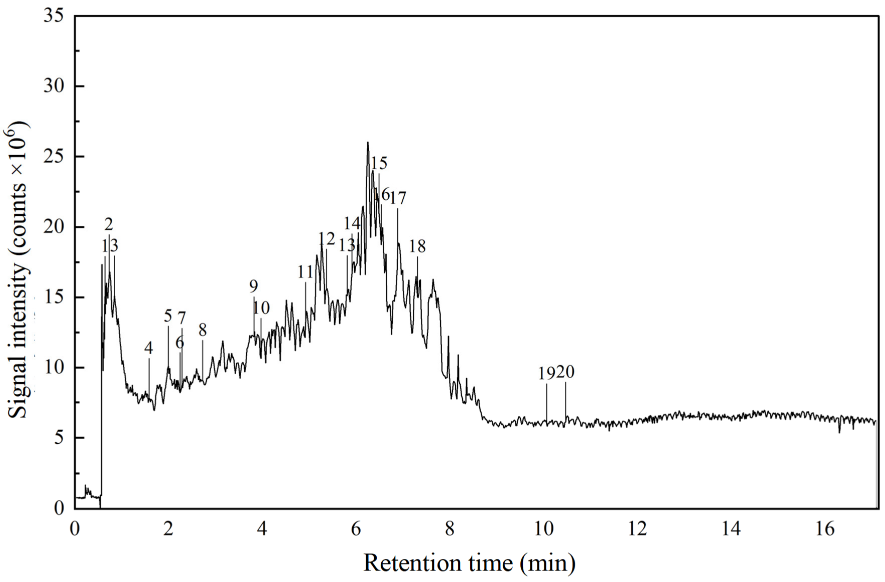

Figure S1. LC-QTOF-MS total ion chromatogram (TIC) of the sample. Peak numbers correspond to compounds listed in Table S1.

Table S1：LC-MS Qualitative Identification Results of Common Allelochemicals.

Serial Number	Retention Time	Molecular Formula	M/Z	Mass Deviation /ppm	Compound Name	Category	Matching Score
1	0.645	$C_7H_{12}O_6$	191.0560	−0.33	D-(-)-Quinic acid	Organic acid	99.86
2	0.732	$C_4H_6O_5$	133.0142	−0.56	Malic acid	Organic acid	99.9
3	0.849	$C_9H_{13}NO_2$	166.0875	0.72	Synephrine	Alkaloid	99.35
4	1.584	$C_7H_6O_4$	153.0194	0.47	Protocatechuic acid	Phenolic acid	99.24
5	1.994	$C_7H_6O_3$	137.0243	−0.59	p-hydroxybenzoic acid	Phenolic acid	99.76
6	2.244	$C_7H_6O_2$	121.0295	−0.17	Benzoic Acid	Phenolic acid	99.98
7	2.288	$C_8H_8O_4$	167.0350	0.14	Vanillic acid	Phenolic acid	98.87
8	2.731	$C_8H_8O_5$	183.0298	−0.30	Methyl gallate	Phenolic acid	97.08
9	3.823	$C_9H_8O_3$	163.0400	−0.26	p-Coumaric acid	Phenolic acid	98.01
10	1.332	$C_7H_{10}O_5$	173.0455	−0.22	Shikimic acid	Organic acid	98.22
11	4.927	$C_{27}H_{32}O_{15}$	595.1666	−0.48	Neoeriocitrin	Flavonoid	98.74
12	5.370	$C_{27}H_{32}O_{14}$	579.1717	−0.45	Naringin	Flavonoid	99.41
13	5.813	$C_{28}H_{34}O_{15}$	609.1821	−0.68	Neohesperidin	Flavonoid	99.16
14	5.916	$C_{21}H_{20}O_{11}$	447.0929	−0.89	Quercetin 7-rhamnoside	Flavonoid	97.69
15	6.494	$C_{16}H_{14}O_6$	301.0716	−0.47	Hesperetin	Flavonoid	83.96
16	6.539	$C_{21}H_{20}O_{12}$	463.0879	−0.68	Isoquercitrin	Flavonoid	95.64
17	6.509	$C_{27}H_{30}O_{16}$	609.1461	−0.02	Kaempferol 3-O-sophoroside	Flavonoid	99.73
18	2.746	$C_{10}H_8O_3$	175.0401	0.07	7-Methoxycoumarin	Coumarins	98.18
19	10.067	$C_{14}H_{28}O_2$	227.2018	0.43	Myristic acid	Fatty acid	99.77
20	10.471	$C_{18}H_{32}O_2$	279.2328	−0.63	Linoleic acid	Fatty acid	97.83

Section S2. MS/MS Spectra of High-Confidence Compounds (Score >80)

Figure S2. MS/MS spectrum of D-(-)-Quinic acid (Peak 1 in Figure S1, Table S1)

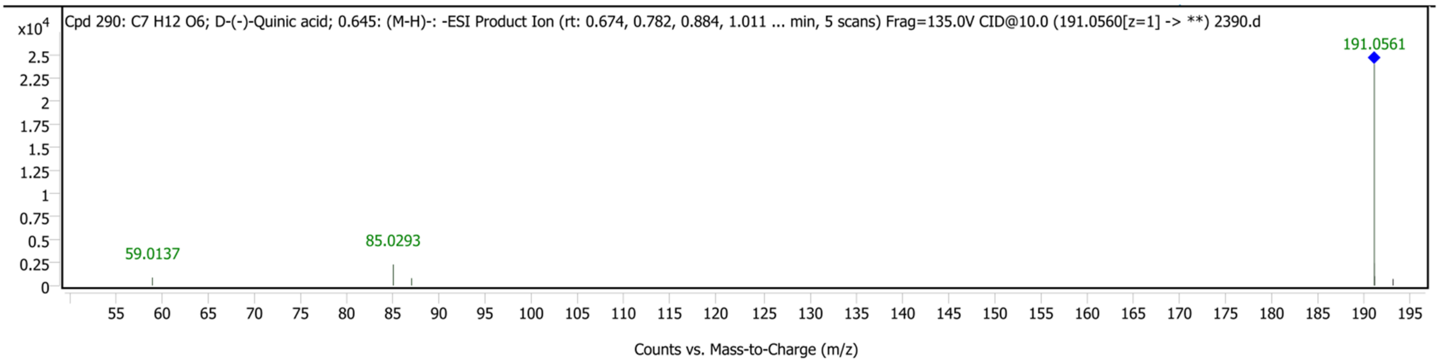

m/z	Z	Abund	Formula	Ion Species	Loss Formula	Loss Mass	Ion Type	Diff (ppm)
59.0137	1	866	C2 H3 O2	M-	C5H8O4	132.0423	Fragment Ion	-2.00
85.0293	1	2277	C4 H5 O2	M-	C3H6O4	106.0266	Fragment Ion	-2.15
87.0082	1	797	C3 H3 O3	M-	C4H8O3	104.0473	Fragment Ion	-6.79
191.0561	1	23955	C7 H11 O6	M-			Molecular Ion	-0.09
191.0649	1	2429						
191.0736	1	820						
191.0874	1	1011						
193.0709	1	719						

Figure S3. MS/MS spectrum of Malic acid (Peak 2 in Figure S1, Table S1)

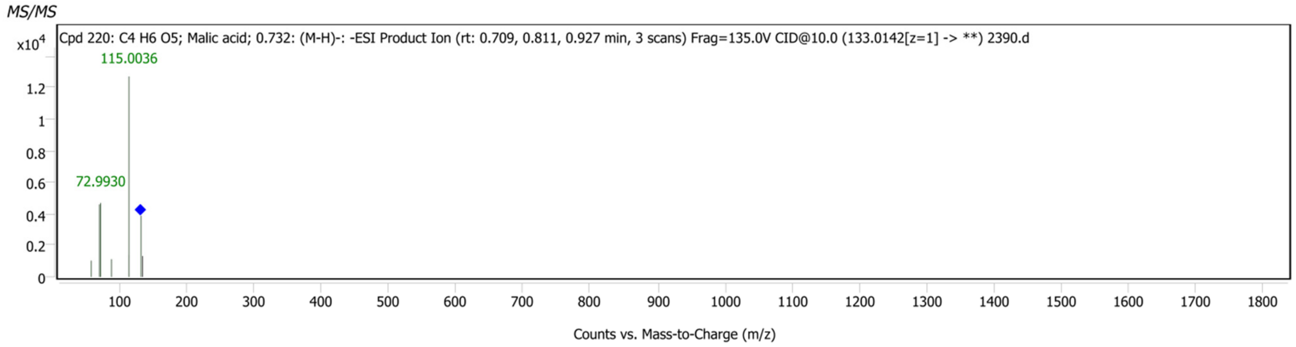

m/z	Z	Abund	Formula	Ion Species	Loss Formula	Loss Mass	Ion Type	Diff (ppm)
59.0140	1	1034	C2 H3 O2	M-	C2H2O3	74.0004	Fragment Ion	2.85
71.0138	1	4599	C3 H3 O2	M-	CH2O3	62.0004	Fragment Ion	-0.33
72.9930	1	4691	C2 H O3	M-	C2H4O2	60.0211	Fragment Ion	-1.92
89.0246	1	1125	C3 H5 O3	M-	CO2	43.9898	Fragment Ion	1.81
115.0036	1	12721	C4 H3 O4	M-	H2O	18.0106	Fragment Ion	-0.42
133.0144	1	3881	C4 H5 O5	M-			Molecular Ion	1.05
115.0102	1	1398						
135.0299	1	1323						

Figure S4. Extracted ion chromatogram (EIC) and full-scan mass spectrum of Synephrine (Peak 3 in Figure S1, Table S1)

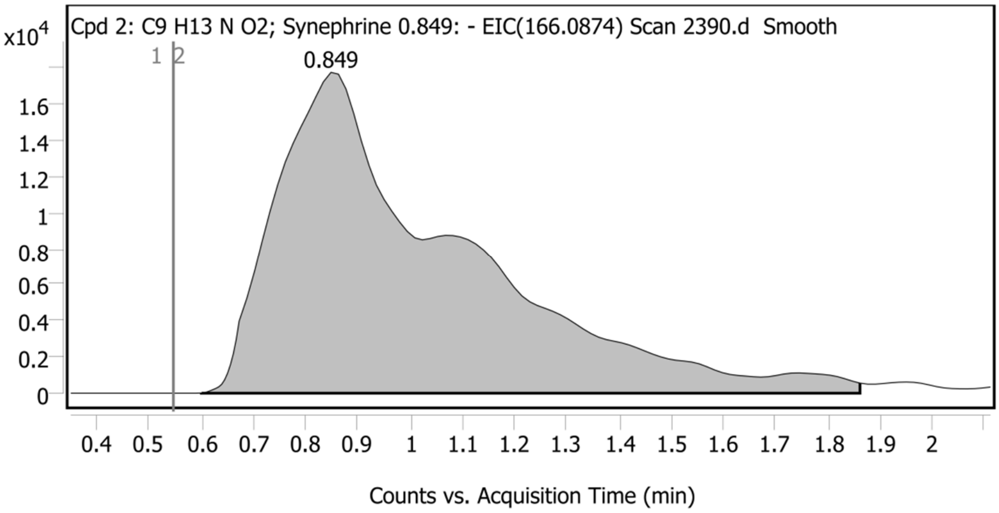

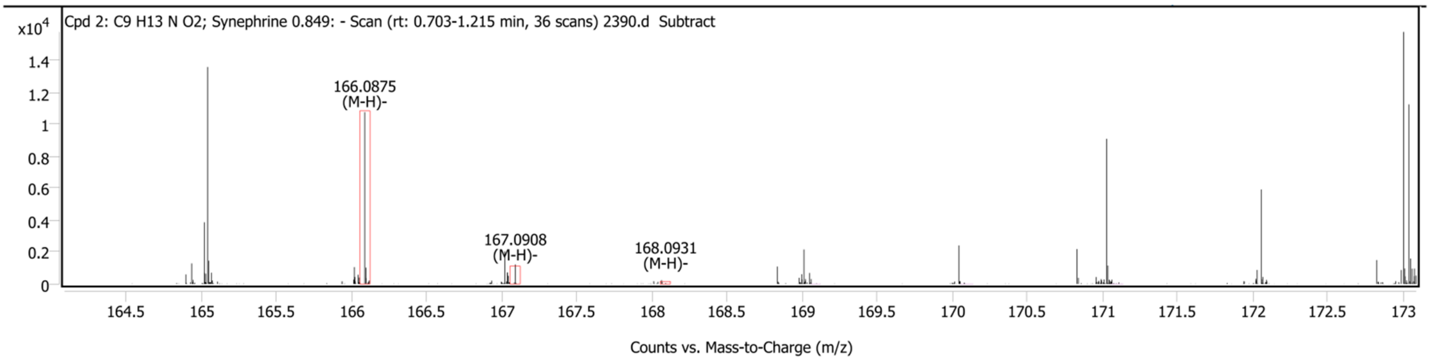

m/z	m/z (Calc)	Diff (ppm)	Abund	Height %	Height % (Calc)	Ion Species	Z
166.0875	166.0874	0.72	10781	100.00	100.00	(M-H)-	1
167.0908	167.0905	1.88	1225	11.36	10.31	(M-H)-	1
168.0931	168.0927	2.40	55	0.51	0.89	(M-H)-	1

Figure S5. Extracted ion chromatogram (EIC) and full-scan mass spectrum of protocatechuic acid (Peak 4 in Figure S1, Table S1)

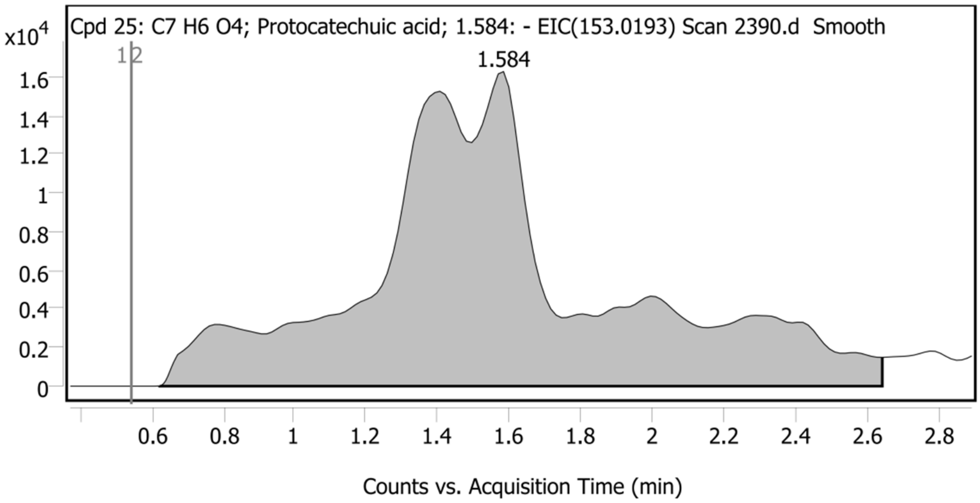

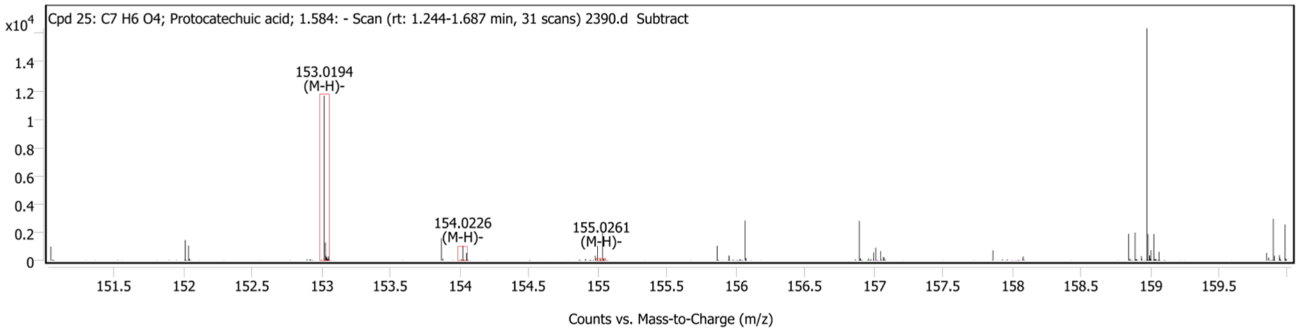

m/z	m/z (Calc)	Diff (ppm)	Abund	Height %	Height % (Calc)	Ion Species	Z
153.0194	153.0193	0.47	11641	100.00	100.00	(M-H)-	1
154.0226	154.0227	-0.56	1045	8.97	7.78	(M-H)-	1
155.0261	155.0242	12.61	65	0.56	1.08	(M-H)-	1

Figure S6. Extracted ion chromatogram (EIC) and full-scan mass spectrum of p-hydroxybenzoic acid (Peak 5 in Figure S1, Table S1)

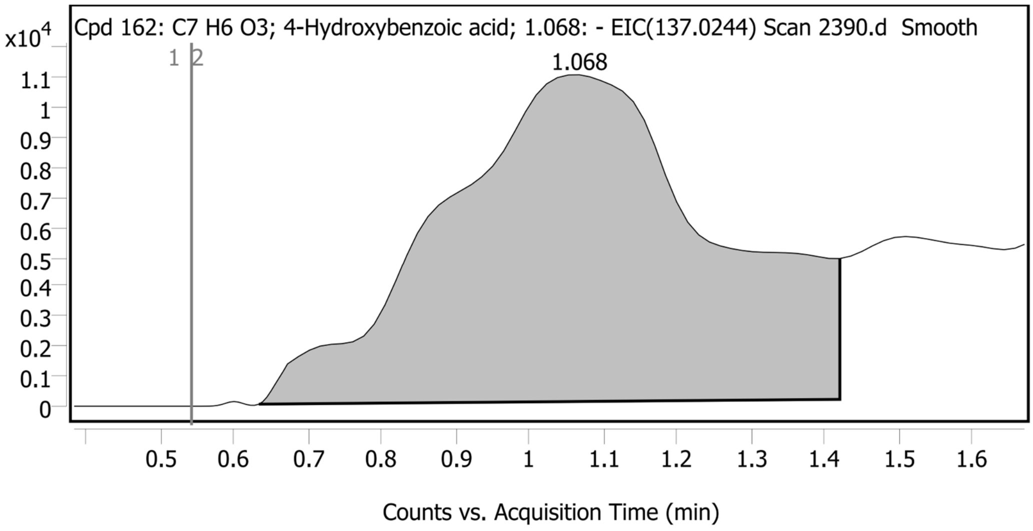

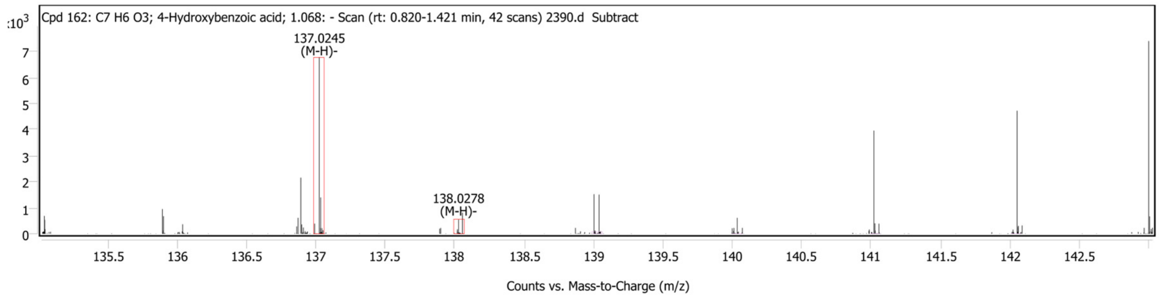

m/z	m/z (Calc)	Diff (ppm)	Abund	Height %	Height % (Calc)	Ion Species	Z
137.0245	137.0244	0.57	6785	100.00	100.00	(M-H)-	1
138.0278	138.0278	0.16	545	8.04	7.74	(M-H)-	1

Figure S7. MS/MS spectrum of Benzoic Acid (Peak 6 in Figure S1, Table S1)

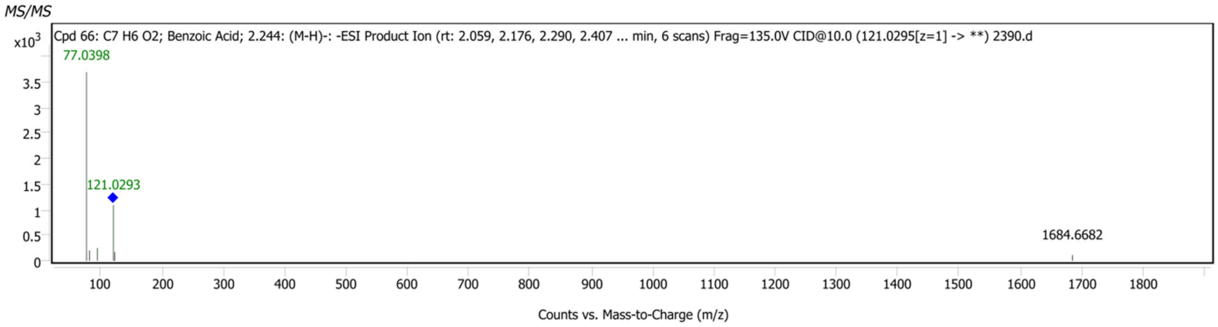

m/z	Z	Abund	Formula	Ion Species	Loss Formula	Loss Mass	Ion Type	Diff (ppm)
77.0398	1	3690	C6 H5	M-	CO2	43.9898	Fragment Ion	1.31
95.0138	1	251	C5 H3 O2	M-	C2H2	26.0157	Fragment Ion	-0.61
121.0293	1	1092	C7 H5 O2	M-			Molecular Ion	-1.57
77.0445	1	127						
77.0461	1	163						
82.0298	1	202						
121.0374	1	142						
123.0091	1	173						
1684.6682	1	113						

Figure S8. MS/MS spectrum of Vanillic acid (Peak 7 in Figure S1, Table S1)

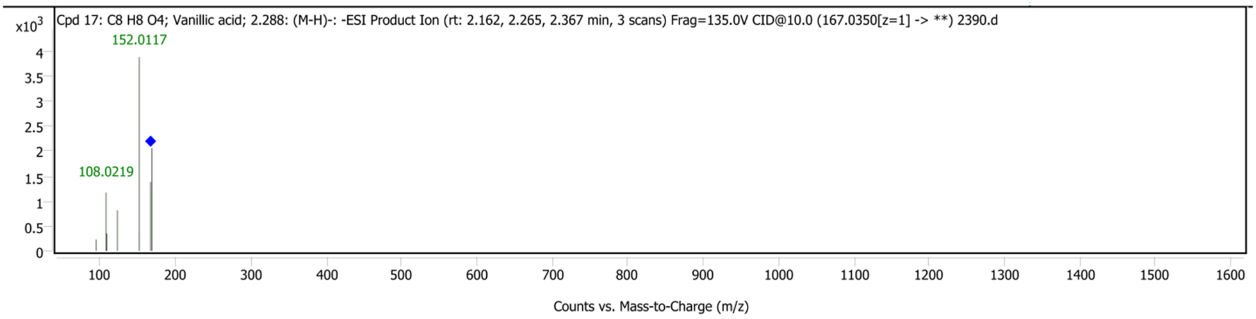

m/z	Z	Abund	Formula	Ion Species	Loss Formula	Loss Mass	Ion Type	Diff (ppm)
95.0145	1	229	C5 H3 O2	M-	C3H4O2	72.0211	Fragment Ion	6.50
108.0219	1	1169	C6 H4 O2	M-	C2H3O2	59.0133	Fragment Ion	2.28
123.0449	1	814	C7 H7 O2	M-	CO2	43.9898	Fragment Ion	-2.46
152.0117	1	3873	C7 H4 O4	M-	CH3	15.0235	Fragment Ion	1.40
167.0359	1	1382	C8 H7 O4	M-			Molecular Ion	5.35
109.0247	1	350						
152.0185	1	383						
168.8369	1	2057						

Figure S9. Extracted ion chromatogram (EIC) and full-scan mass spectrum of Methyl gallate (Peak 8 in Figure S1, Table S1)

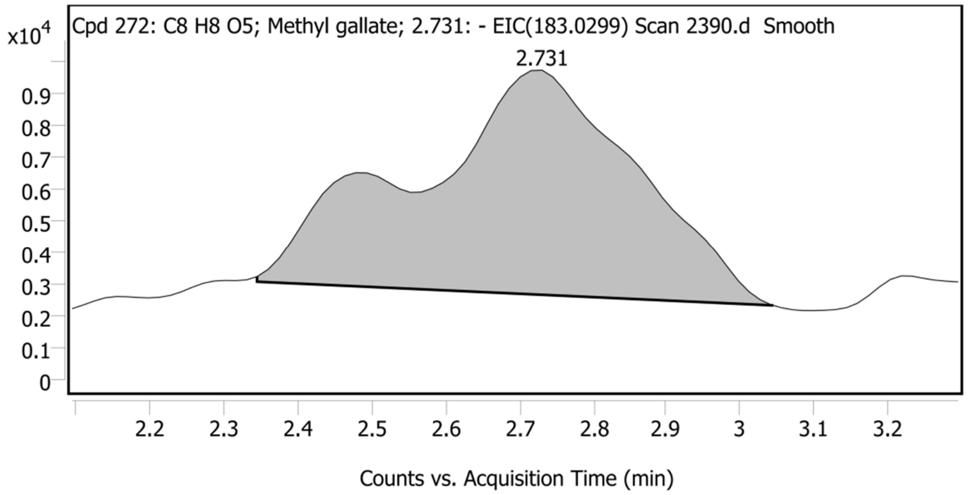

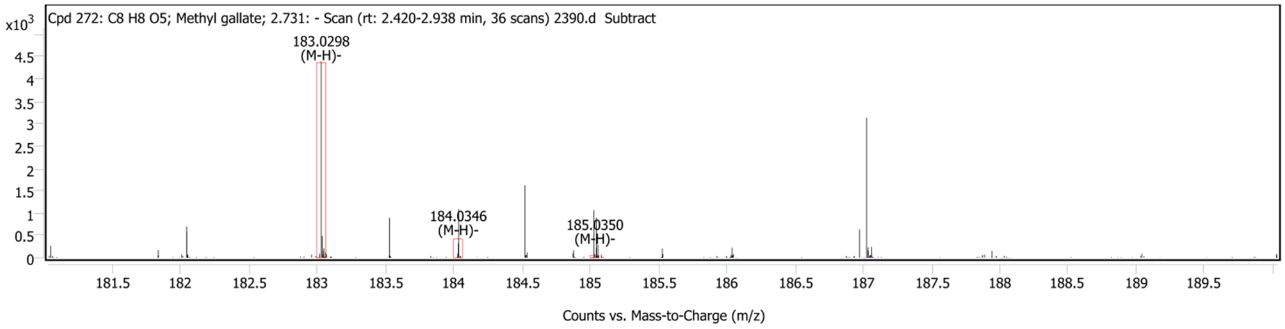

m/z	m/z (Calc)	Diff (ppm)	Abund	Height %	Height % (Calc)	Ion Species	Z
183.0298	183.0299	-0.30	4394	100.00	100.00	(M-H)-	1
184.0346	184.0333	6.95	323	7.35	8.92	(M-H)-	1
185.0350	185.0348	0.93	86	1.96	1.38	(M-H)-	1

Figure S10. Extracted ion chromatogram (EIC) and full-scan mass spectrum of p-Coumaric acid (Peak 9 in Figure S1, Table S1)

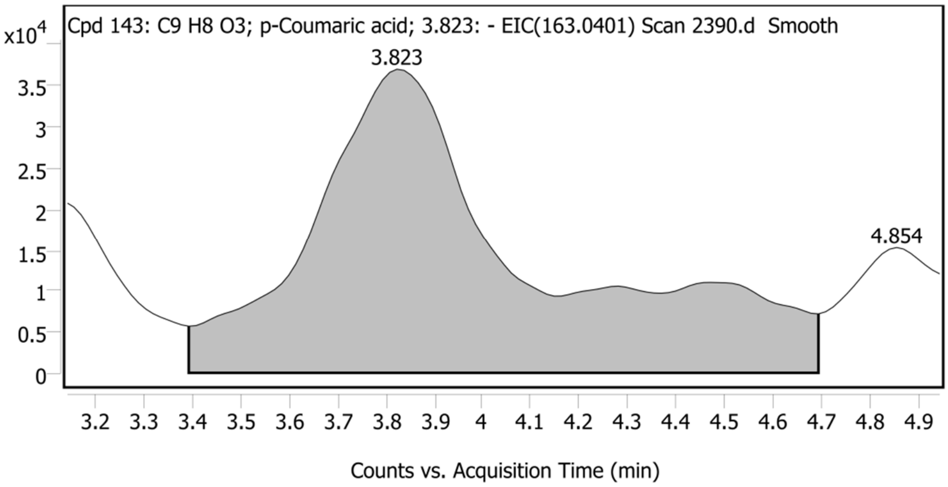

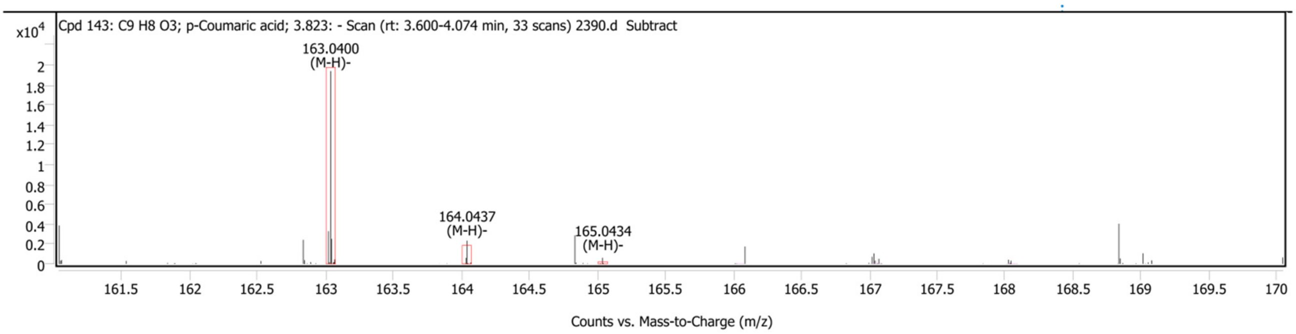

m/z	m/z (Calc)	Diff (ppm)	Abund	Height %	Height % (Calc)	Ion Species	Z
163.0400	163.0401	-0.26	19400	100.00	100.00	(M-H)-	1
164.0437	164.0435	1.64	2340	12.06	9.93	(M-H)-	1
165.0434	165.0454	-12.21	72	0.37	1.06	(M-H)-	1

Figure S11. Extracted ion chromatogram (EIC) and full-scan mass spectrum of Shikimic acid (Peak 10 in Figure S1, Table S1)

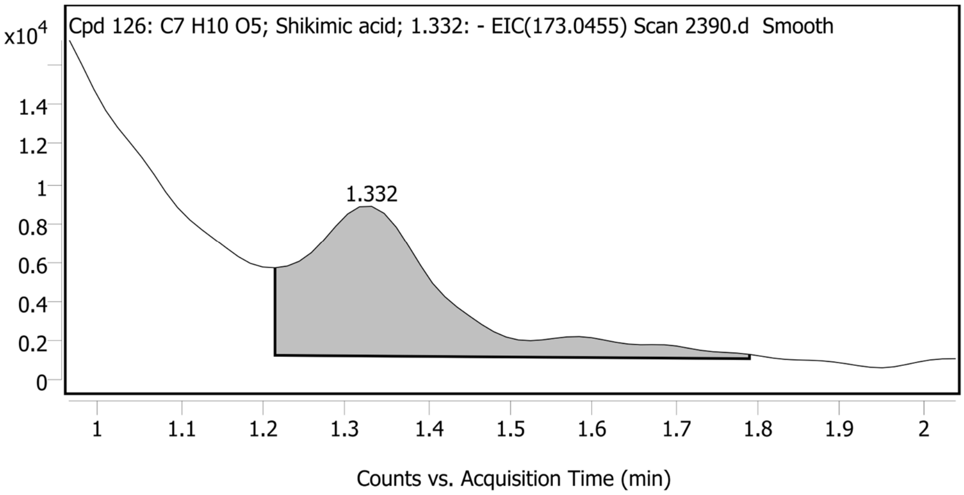

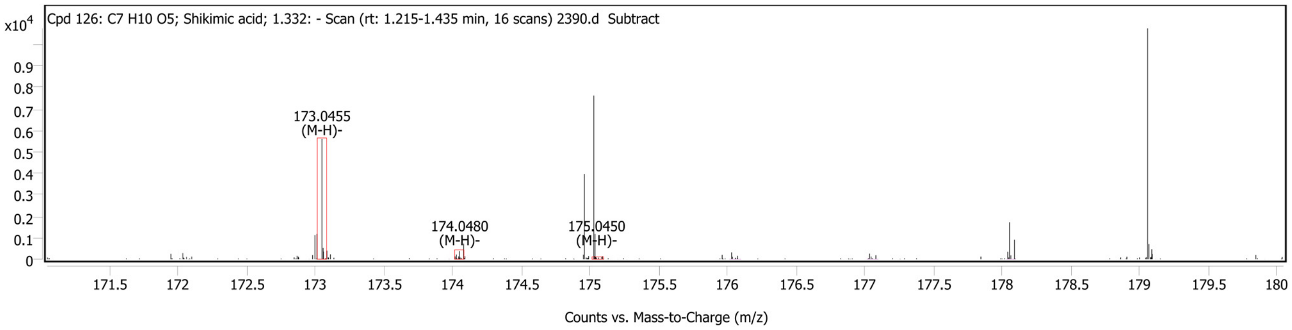

m/z	m/z (Calc)	Diff (ppm)	Abund	Height %	Height % (Calc)	Ion Species	Z
173.0455	173.0455	-0.22	5601	100.00	100.00	(M-H)-	1
174.0480	174.0490	-5.34	379	6.76	7.86	(M-H)-	1
175.0450	175.0503	-30.29	72	1.28	1.30	(M-H)-	1

Figure S12. MS/MS spectrum of Neoeriocitrin (Peak 11 in Figure S1, Table S1)

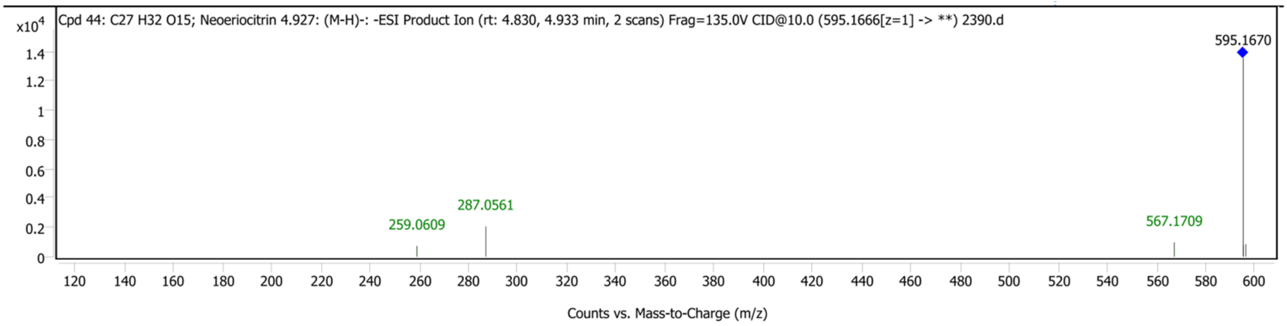

m/z	Z	Abund	Formula	Ion Species	Loss Formula	Loss Mass	Ion Type	Diff (ppm)
259.0609	1	733	C14 H11 O5	M-	C13H20O10	336.1056	Fragment Ion	-1.14
287.0561	1	2076	C15 H11 O6	M-	C12H20O9	308.1107	Fragment Ion	-0.12
567.1709	1	976	C26 H31 O14	M-	CO	27.9949	Fragment Ion	-1.90
595.1670	1	13568						
595.1847	1	822						
595.2229	1	770						
596.1862	1	862						

Figure S13. Extracted ion chromatogram (EIC) and full-scan mass spectrum of Naringin (Peak 12 in Figure S1, Table S1)

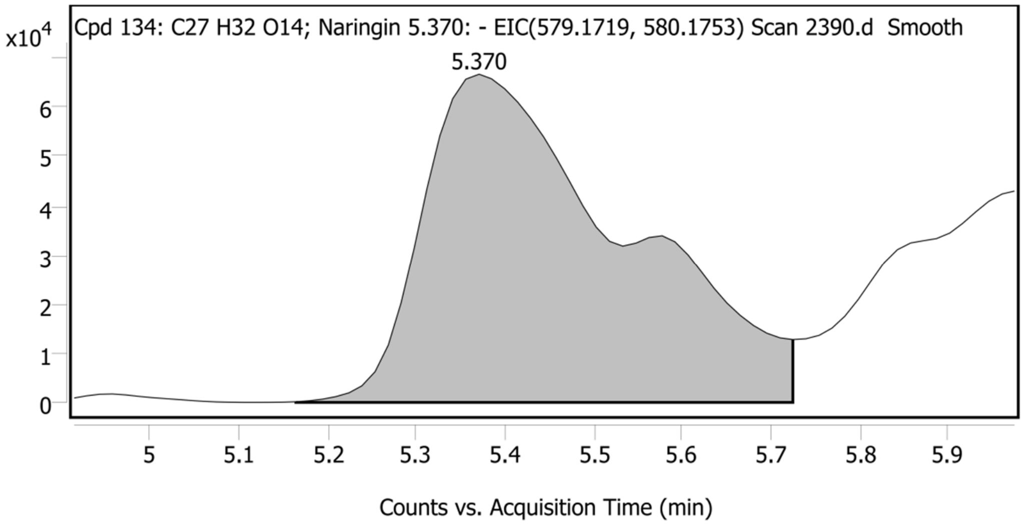

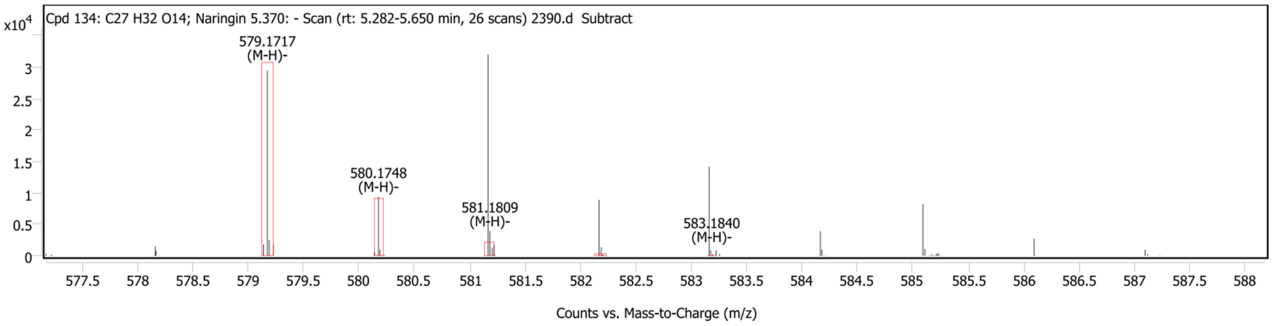

m/z	m/z (Calc)	Diff (ppm)	Abund	Height %	Height % (Calc)	Ion Species	Z
579.1717	579.1719	-0.45	29438	100.00	100.00	(M-H)-	1
580.1748	580.1753	-0.87	9400	31.93	30.09	(M-H)-	1
581.1809	581.1777	5.45	3921	13.32	7.25	(M-H)-	1
583.1840	582.1804	1723.80	71	0.24	1.27	(M-H)-	1

Figure S14. Extracted ion chromatogram (EIC) and full-scan mass spectrum of Neohesperidin (Peak 13 in Figure S1, Table S1)

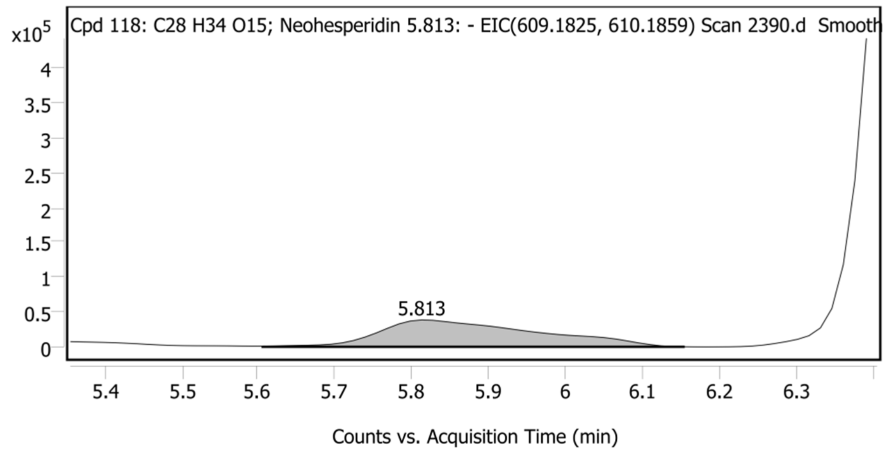

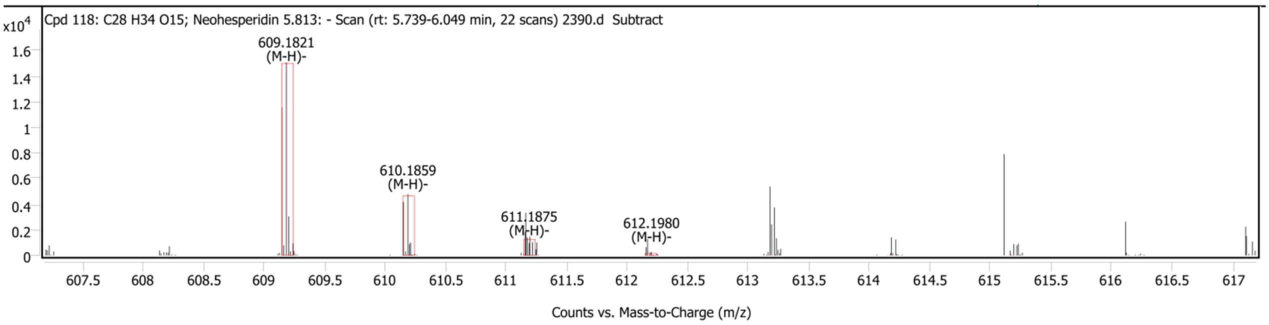

m/z	m/z (Calc)	Diff (ppm)	Abund	Height %	Height % (Calc)	Ion Species	Z
609.1821	609.1825	-0.68	15101	100.00	100.00	(M-H)-	1
610.1859	610.1859	-0.08	4794	31.74	31.23	(M-H)-	1
611.1875	611.1883	-1.29	971	6.43	7.80	(M-H)-	1
612.1980	612.1910	11.50	283	1.87	1.42	(M-H)-	1

Figure S15. Extracted ion chromatogram (EIC) and full-scan mass spectrum of Quercetin 7-rhamnoside (Peak 14 in Figure S1, Table S1)

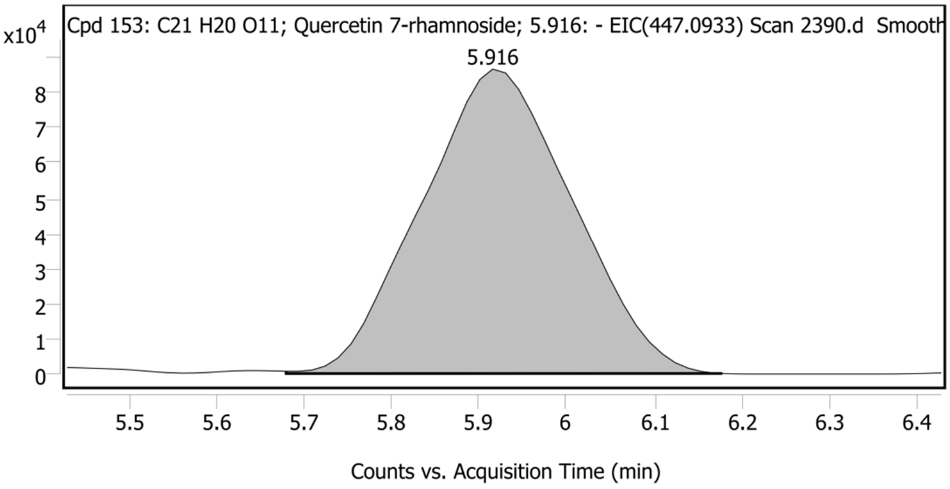

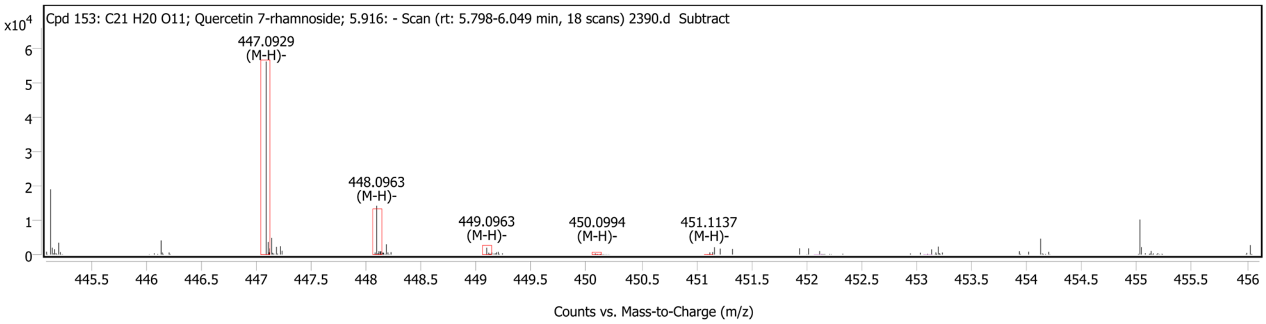

m/z	m/z (Calc)	Diff (ppm)	Abund	Height %	Height % (Calc)	Ion Species	Z
447.0929	447.0933	-0.89	56001	100.00	100.00	(M-H)-	1
448.0963	448.0967	-0.87	14152	25.27	23.35	(M-H)-	1
449.0963	449.0989	-5.70	2013	3.59	4.86	(M-H)-	1
450.0994	450.1016	-4.82	154	0.28	0.71	(M-H)-	1
451.1137	451.1039	21.69	61	0.11	0.09	(M-H)-	1

Figure S16. Extracted ion chromatogram (EIC) and full-scan mass spectrum of Hesperetin (Peak 15 in Figure S1, Table S1)

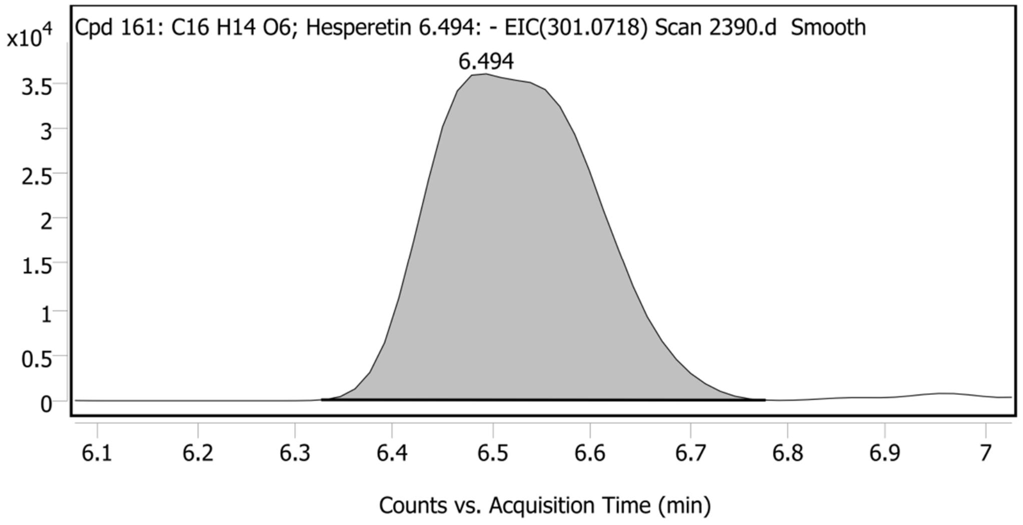

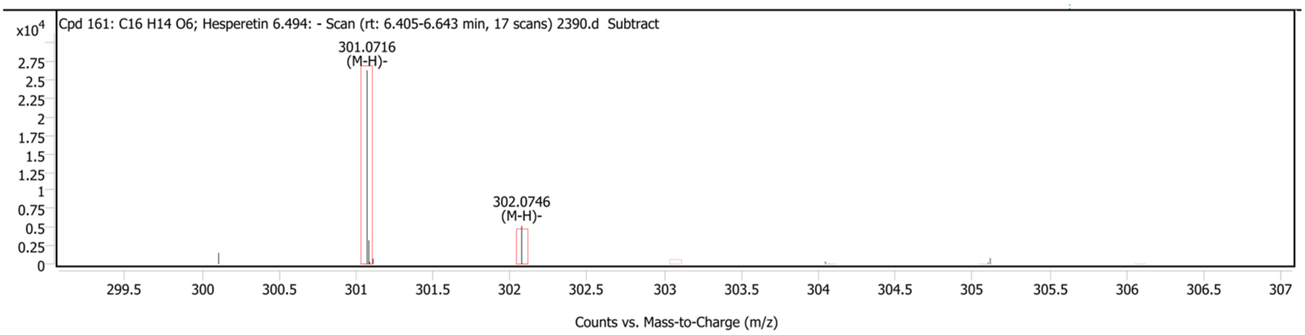

m/z	m/z (Calc)	Diff (ppm)	Abund	Height %	Height % (Calc)	Ion Species	Z
301.0716	301.0718	-0.47	26375	100.00	100.00	(M-H)-	1
302.0746	302.0752	-1.70	5180	19.64	17.68	(M-H)-	1

Figure S17. Extracted ion chromatogram (EIC) and full-scan mass spectrum of Isoquercitrin (Peak 16 in Figure S1, Table S1)

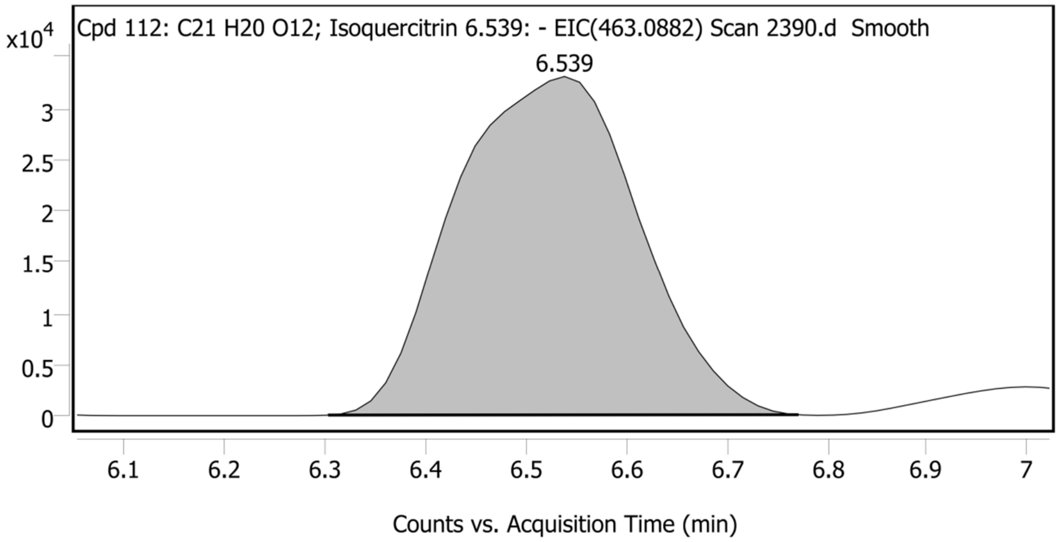

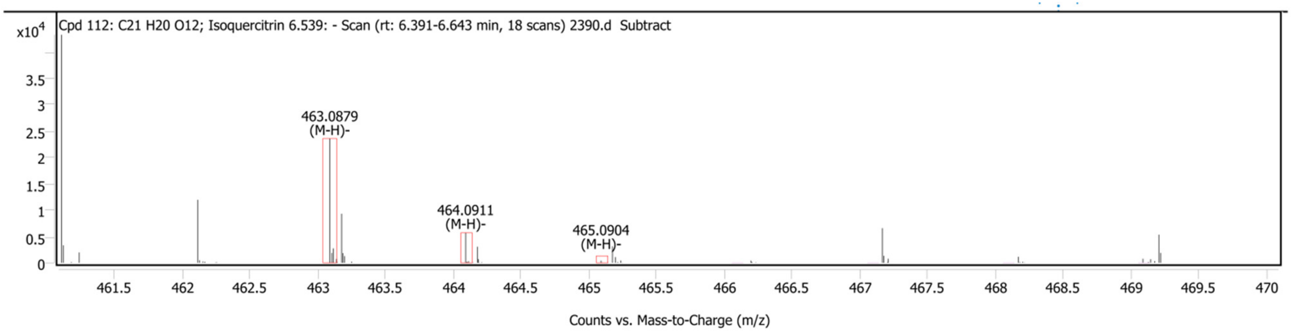

m/z	m/z (Calc)	Diff (ppm)	Abund	Height %	Height % (Calc)	Ion Species	Z
463.0879	463.0882	-0.68	23552	100.00	100.00	(M-H)-	1
464.0911	464.0916	-1.09	5743	24.39	23.39	(M-H)-	1
465.0904	465.0938	-7.24	422	1.79	5.08	(M-H)-	1

Figure S18. Extracted ion chromatogram (EIC) and full-scan mass spectrum of Kaempferol 3-O-sophoroside (Peak 17 in Figure S1, Table S1)

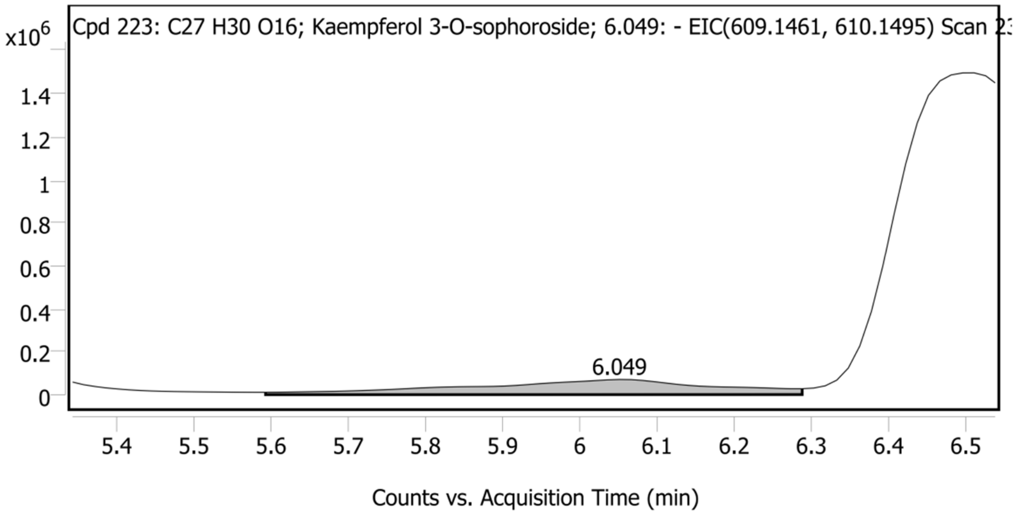

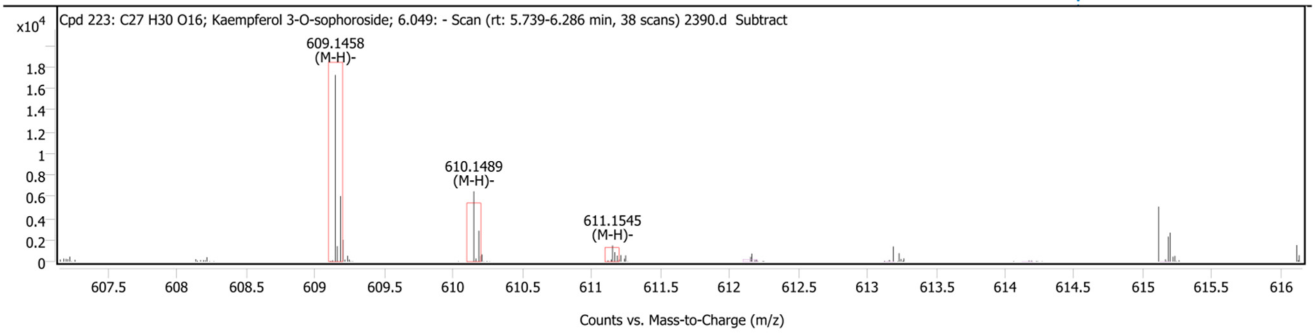

m/z	m/z (Calc)	Diff (ppm)	Abund	Height %	Height % (Calc)	Ion Species	Z
609.1458	609.1461	-0.45	17238	100.00	100.00	(M-H)-	1
610.1489	610.1495	-0.98	6497	37.69	30.15	(M-H)-	1
611.1545	611.1518	4.37	1499	8.69	7.67	(M-H)-	1

Figure S19. Extracted ion chromatogram (EIC) and full-scan mass spectrum of 7-Methoxycoumarin (Peak 18 in Figure S1, Table S1)

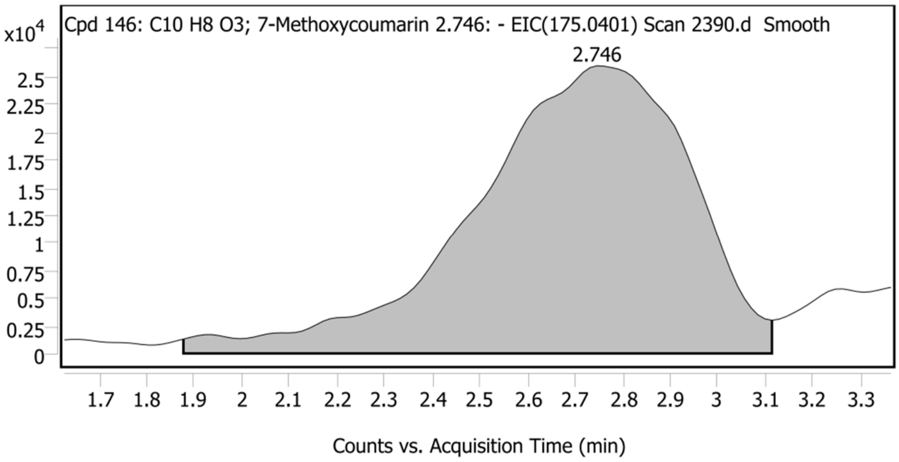

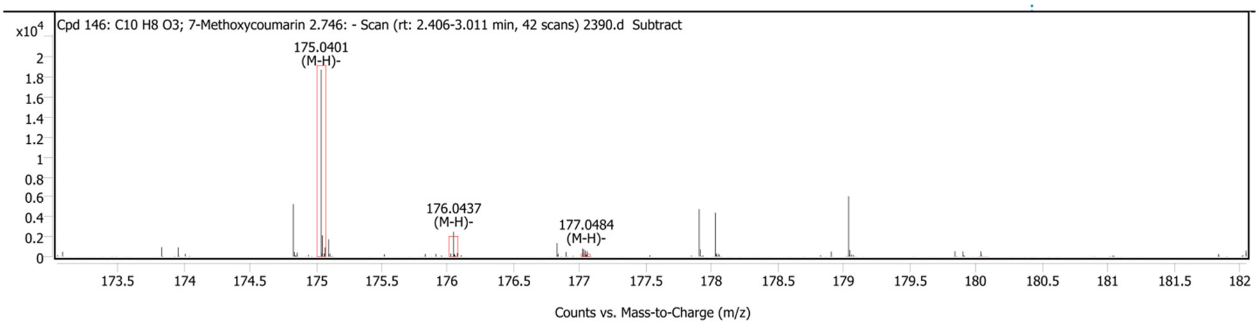

m/z	m/z (Calc)	Diff (ppm)	Abund	Height %	Height % (Calc)	Ion Species	Z
175.0401	175.0401	0.07	18739	100.00	100.00	(M-H)-	1
176.0437	176.0435	1.46	2476	13.21	11.01	(M-H)-	1
177.0484	177.0455	16.32	151	0.81	1.16	(M-H)-	1

Figure S20. MS/MS spectrum of Myristic acid (Peak 19 in Figure S1, Table S1)

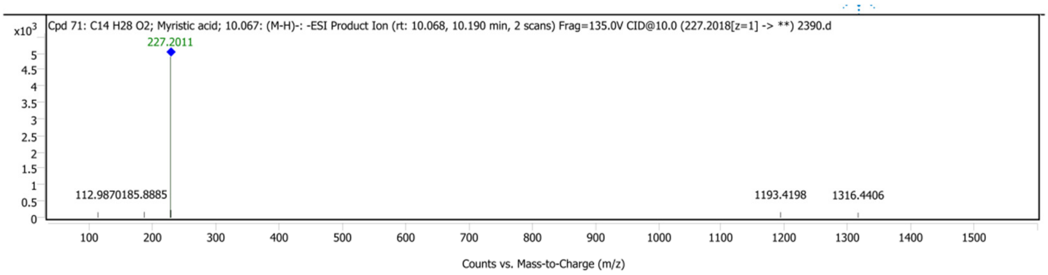

m/z	Z	Abund	Formula	Ion Species	Loss Formula	Loss Mass	Ion Type	Diff (ppm)
227.2011	1	4891	C14 H27 O2	M-			Molecular Ion	-2.26
112.9870	1	168						
185.8885	1	177						
227.2123	1	250						
227.2363	1	214						
228.2129	1	234						
1193.4198	1	169						
1316.4406	1	155						

Figure S21. Extracted ion chromatogram (EIC) and full-scan mass spectrum of Linoleic acid (Peak 20 in Figure S1, Table S1)

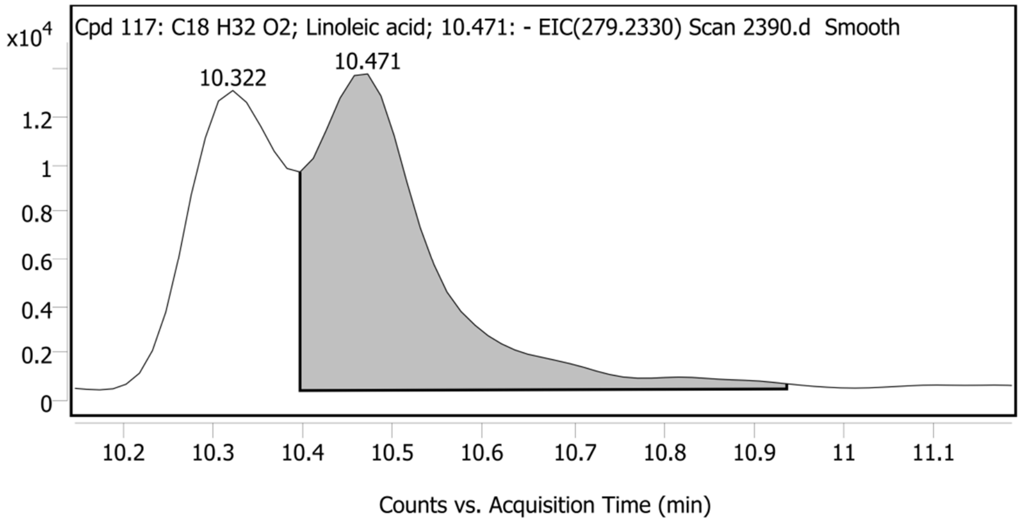

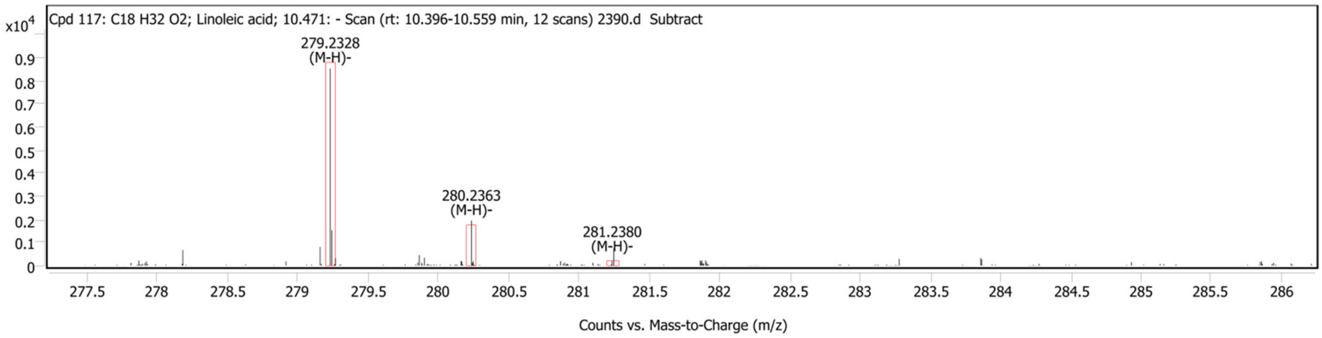

m/z	m/z (Calc)	Diff (ppm)	Abund	Height %	Height % (Calc)	Ion Species	Z
279.2328	279.2330	-0.63	8521	100.00	100.00	(M-H)-	1
280.2363	280.2364	-0.31	1949	22.87	19.90	(M-H)-	1
281.2380	281.2393	-4.57	197	2.31	2.29	(M-H)-	1
